# An upside to chronic inflammation? Starvation reconfigures immune pathways in *Manduca sexta*, producing chronic inflammation that may help defend against bacterial infection

**DOI:** 10.1371/journal.pone.0358460

**Published:** 2026-09-21

**Authors:** Shelley A. Adamo, Madeleine R. Burns, Megan H. Englehardt, Arisha Grabtchak, Rose Kearney, Claire A. E. Martin, Maya R. Richmond

**Affiliations:** Department of Psychology and Neuroscience, Dalhousie University, Halifax, Nova Scotia, Canada; University of California Riverside, UNITED STATES OF AMERICA

## Abstract

Most animals face periods of food scarcity, and pathogens are ever-present. Therefore, selection will favour animals that can maintain immune defense during starvation, despite declining resources. Using the caterpillar *Manduca sexta* as a model system, we show how two days of starvation reconfigures the immune system. Metabolomic and proteomic analysis of the plasma showed that starvation shifted the immune system towards a more ‘pro-inflammatory’ state, with increased activity in the phenoloxidase/eumelanin pathways relative to controls. Melanization occurred more rapidly in starved caterpillars, demonstrating that these molecular changes had functional significance. This chronic inflammation correlated with a decline in the abundance of the important antioxidant glutathione (GSH) in the plasma. Increasing GSH in the plasma either by injection or diet reduced melanization in starved caterpillars, suggesting that the decline in GSH abundance could contribute to the increase in inflammation. Although starvation enhanced phenoloxidase pathway activity, it also reduced nodulation, suggesting a decline in cell-mediated immunity. Nevertheless, we found that starved caterpillars had fewer bacteria in their hemolymph (i.e., colony forming units, CFUs) 3 h after infection with live *Bacillus cereus* than did fed controls or weight-matched controls. However, increasing GSH in starved caterpillars increased mortality from *B. cereus* infection. We suggest that the upregulation of the phenoloxidase/eumelanin pathways during starvation is beneficial because it partially compensates for concurrent declines in other immune components. Without this chronic inflammation, our results suggest that caterpillars would experience an even greater decline in disease resistance during starvation.

## 1 Introduction

Immune systems are energetically expensive in insects (e.g., [1]), but necessary to combat potentially lethal pathogens. However, their activation results in damage (e.g., [2]), leading to a decrease in both lifespan ([2]) and reproduction ([3]). How insects, and other organisms, balance the costs and benefits of immune defence remain unclear ([4]). This problem is unlikely to have a single optimal solution for any animal, as the optimal solution will vary depending on a number of factors including current internal and external conditions. The need to shift the relative balance between immune activity and immunopathology under different conditions ([5]) helps explain the evolution of the complex regulatory networks controlling insect immunity (e.g., the phenoloxidase pathway, [6]). Nevertheless, some immune responses seem ‘dysregulated’, leading to phenomena such as immune-induced declines in muscle capacity, immune-mediated insulin resistance, and tissue damage from chronic inflammation in both insects ([2,7]) and mammals ([8,9]). However, a re-examination of immune effects on muscle (e.g., [10,11]) and insulin resistance ([12]) in insects has shown that, under some conditions, these are not pathologies, but are beneficial given the animal’s circumstances. In this paper we examine whether chronic inflammation could also be beneficial in the model insect *Manduca sexta*.

Chronic inflammation in insects is characterized by persistent immune activation, usually accompanied by tissue damage (e.g., [13–15]). Chronic inflammation is assumed to be the product of immune dysregulation in both mammals ([9]) and insects (e.g., [2,13,15]), meaning that the underlying immune pathways are no longer appropriately controlled. In mammals, chronic inflammation is involved in a number of disease processes, demonstrating that it can be pathological (e.g. Alzheimer’s, [16]). However, chronic inflammation can also be induced by environmental conditions, such as an inadequate diet in mammals ([17,18]). Inflammation caused by food scarcity is considered chronic inflammation because it is not triggered by a pathogen or tissue damage, and, therefore, is considered an inappropriate activation ([9,17]). Starvation is thought to cause chronic inflammation because resource loss leads to a failure within the immune system’s regulatory network ([17]). Insects (e.g., the caterpillar *M. sexta*) also exhibit chronic inflammation during periods of low food availability ([19]). Almost all animals experience periods of low food availability and, therefore, natural selection will favour individuals who can survive some starvation. Given these selective forces, it seems reasonable to examine whether the chronic inflammation produced by food limitation serves some function. Other phenomena, such as fever and illness-induced anorexia were long thought to be pathologies, but we now know that they can be adaptive responses under some conditions ([20]). However, chronic inflammation has not been studied from this perspective.

Although insects lack the equivalent of acquired immunity, they do have something similar to vertebrate innate immunity ([21]). Traditionally, insect immunity has been described as having two arms: humoral and cell-mediated immunity ([22,23]). An immune response begins with the recognition of pathogen-specific molecular motifs (e.g., peptidoglycans) by specialized proteins in the hemolymph (i.e., pathogen recognition molecules, [23]). Once a pathogen is recognized, a series of biochemical reactions is initiated that results in the activation of cytokines ([23]), and the generation of pathogen-killing reactive molecules via the phenoloxidase (PO) pathway ([6]). Cytokines also induce hemocytes (insect blood cells) and the fat body (an insect immune organ) to produce antimicrobial peptides and proteins ([22–24]). These blood-borne molecules are part of the insect humoral immune system. Cytokines also help activate cell-mediated immunity, resulting in phagocytosis, nodulation and/or encapsulation ([25,26]). Immune components are interconnected in insects (e.g., [27]), allowing them to work together to eliminate pathogens from the insect body. It is an efficient and robust system and has allowed insects to flourish even in pathogen-ridden environments (e.g., dung beetles).

**Fig 1:**
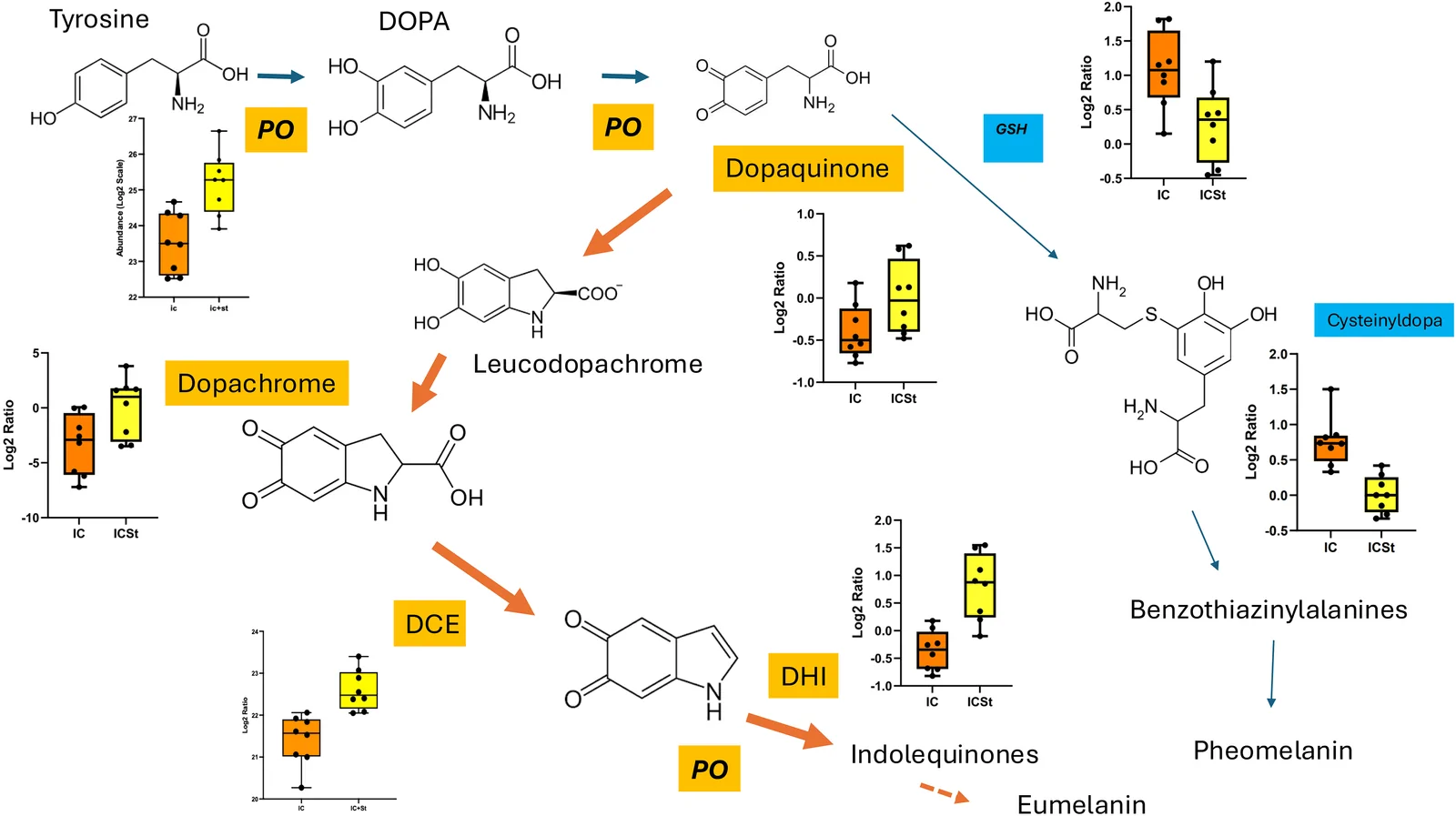
Simplified schematic of the PO-catalyzed eumelanin/pheomelanin pathways. Orange squares denote compounds that increase in abundance during an immune challenge under starvation conditions (ICSt) compared with an immune challenge in fed animals (IC). A blue rectangle denotes compounds that decrease in abundance during ICSt relative to IC. When GSH abundance is low (i.e., during starvation) dopaquinone is recruited into the eumelanin pathway, leading to the generation of reactive molecules ([28]). DCE – dopachrome conversion enzyme, DHI – 5,6-dihydroxyindole, GSH – glutathione, PO – phenoloxidase, The bar graphs illustrate the significant changes in abundance in ICSt relative to IC. The y-axis represents the log_2_ ratio of the peak intensity ratio (i.e., 12C-samples/13C-reference) for the metabolites. For the proteins, the y-axis represents the abundance of each protein transformed to a log_2_ scale. The central line represents the median, and the top and bottom of the bar represents the 25th and 75th percentiles. The error bars denote minimum and maximum values. Dots represent individual samples. N = 8/group for all graphs except PO and DCE (n = 10/group). Pathways adapted from Nappi et al. [29] and Dolezal [28].

One of the most powerful immune components in the insect is the phenoloxidase (PO) pathway ([6]). The PO enzyme exists in an inactive pro-form in the hemolymph ([6]). It is activated by a serine proteinase that is at the end of a cascade of serine proteinases ([6]). Each of these proteinases is heavily regulated by serine protease inhibitors (i.e., serpins, [6]). Once activated, PO catalyzes a series of reactions in the eumelanin pathway (Fig 1). These reactions lead to the formation of melanin and the production of reactive molecules and antimicrobial compounds, such as 5,6-dihydroxyindole ([28]). 5,6-dihydroxyindole kills pathogens in *M. sexta* ([30,31]). The PO pathway is part of the insect’s first response to infection ([32]) and activation can occur within 10 min in injured *Drosophila* ([6]). It is a vital part of the insect immune system. A decline in PO activity has been shown to increase host susceptibility to bacterial pathogens in *M. sexta* ([33]) and other insects ([6]).

As in vertebrate immune systems, insect immune systems can injure the host (e.g., [2]). The PO pathway is especially damaging. Its activation can harm host tissue and even kill the host ([34]). Increased PO activity is considered an example of ‘inflammation’ in insects (e.g., [2]). As in mammals, when this inflammation becomes chronic, it leads to lifespan-reducing damage ([2]). Given PO’s damage potential, it is not surprising that insects have another mechanism, in addition to serpins, to keep PO activity low. PO-generated inflammation is kept in check in well-fed animals by the antioxidant glutathione (GSH) ([35,36]). GSH can quench the reactive molecules generated by the PO pathway ([6,28]). GSH can also redirect a key substrate (Dopaquinone, Fig 1) towards the competing pheomelanin pathway ([28]).

Food restriction decreases GSH abundance and increases PO activity in *M. sexta* ([19]). The resulting increase in PO activity, with its potential for damage, may represent a pathological cost of starvation. Alternatively, lowering GSH abundance could be an adaptive response if enhanced PO-generated inflammation is beneficial during starvation.

Reduced nutrition typically leads to a decline in disease resistance in insects ([37,38]), but it can also be immunoenhancing against some pathogens ([39,40]), including in *M. sexta* ([19]). PO activity is sensitive to nutrient levels in insects, and this may play a role in the changes in disease resistance with starvation ([37]). However, how reduced nutrition affects PO activity is unknown. It is also unknown, what function, if any, changes in PO activity play in immunity during starvation. In this paper we use metabolomics and proteomics to document the changes in the plasma that occur in *M. sexta* in response to: a) starvation, b) an immune challenge, and c) an immune challenge combined with starvation, relative to untreated controls. Using this information, we describe how the phenoloxidase/eumelanin pathways are altered during starvation to produce chronic inflammation. A detailed pathway analysis is possible because the immune system of larval *M. sexta* has been studied extensively (e.g., [27,41–43]). We then manipulate GSH levels to test whether chronic PO activity benefits *M. sexta* under starvation conditions.

## 2 Materials and methods

### 2.1 Animals

*Manduca sexta* (Linnaeus 1763) caterpillars were collected from our colony. The colony was derived from eggs from Great Lakes Hornworm (Romeo, MI, USA). Animals were maintained as described in Adamo et al. [19]. Caterpillars were reared in individual cups (96 ml, 6 cm diameter and 3.6 cm height) from the first until the fourth instar. Upon molting to the fifth instar, caterpillars were placed in larger cups (473 ml, 8 cm diameter and 11 cm height) that contained stiff black mesh to raise them above the frass. All caterpillars were fed ad-libitum on a high nutrition, wheatgerm-based diet (F9783B; Frontier Agricultural Sciences, Newark, DE, USA), unless otherwise stipulated. Caterpillars were maintained between 22 and 24°C, on a 12 h light/dark cycle, and relative humidity 45–70%.

All experiments were approved by the University Committee on Laboratory Animals at Dalhousie University (Protocol number I23-12).

### 2.2 Treatments

**Fig 2:**
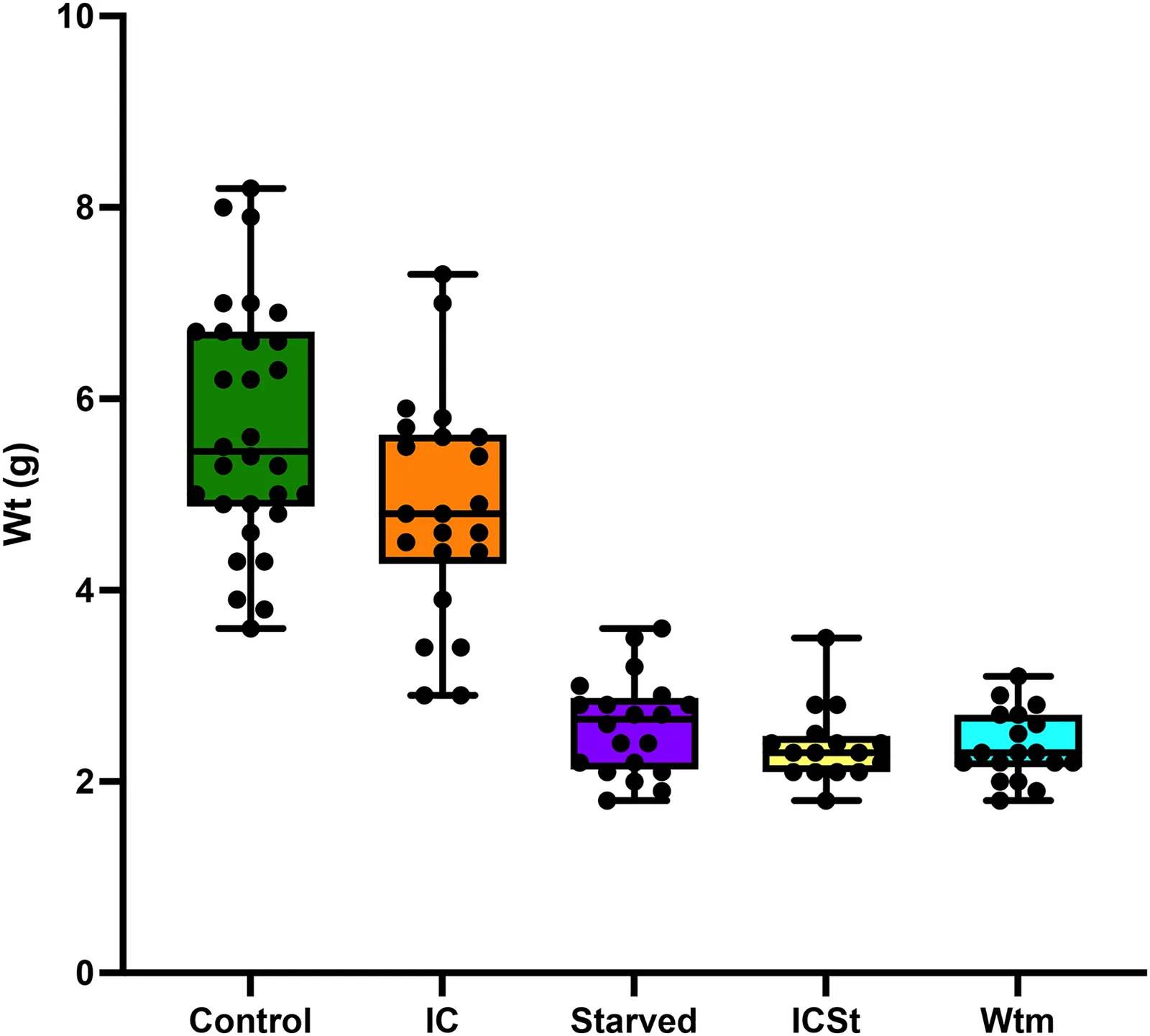
Box plots of caterpillar weight on testing day (5−3). Weight differed significantly across groups (F4, 101)=70.0, p < 0.0001). Control caterpillars were heavier than all other groups (Tukey’s multiple comparison test, p < 0.02). Wtm were not significantly different in weight from the cellulose fed caterpillars (i.e., St and ICSt) (Tukey’s multiple comparison test, p > 0.9). The central bar line denotes the median, with the top of the bar representing the 75th percentile, with the bottom of the bar representing the 25th percentile. Error bars show the maximum and minimum values. Dots represent individual data points. Control n = 30, IC (immune challenged) n = 22; Starved n = 20, ICSt (starved and immune challenged) n = 16, Wtm (weight matched control) n = 18.

To examine the impact of resource limitation on the immune response, caterpillars were randomly assigned into 4 different groups: 1) unhandled controls (Control), 2) immune challenged caterpillars (IC), 3) starved caterpillars (St), and 4) caterpillars that were both starved and given an immune challenge (ICSt). Starved caterpillars were given a wet cellulose diet (50g cellulose/150 mL water) that supplied the animals with water, but not with nutrition. Caterpillars were immune-challenged by injecting them with an equal mix of heat-killed bacteria/fungi (*Bacillus cereus* (Gram positive bacteria), *Serratia marcescens* (Gram-negative bacteria) and *Beauveria bassiana* (fungus)) (see [19] for details). Because of the difference in weights between immune-challenged animals fed the normal diet, and those fed on cellulose (the starvation diet) (Fig 2), the dose of heat-killed pathogens injected into each caterpillar was determined by the caterpillar’s weight that day (10 µL/g caterpillar), unless otherwise stipulated. When it was important to control for size, we included weight-matched controls (Wtm). These caterpillars were the same approximate weight as the starved caterpillars, but were approximately one day younger. These caterpillars were fed only on the high quality control diet. Also, we opted not to use sham-injected controls in these experiments, because sham injections are known to induce an immune response (e.g., [44]), and wounding, in particular, activates PO ([6]). Therefore, our immune-challenged caterpillars received a dual challenge – one from wounding and one from the injection of immunogenic material.

Diet treatments began the day after the caterpillars molted into the 5th instar (i.e., 5th-1). Therefore, all caterpillars had 1 day on the high quality control diet at the beginning of the 5th instar (i.e., Day 5th-0–5th-1). We chose the 5th instar for our treatments because it is during this instar that weight gain is most rapid, and it is the most commonly used instar in other studies on diet effects in *M. sexta* ([19,45–47]). On the following day (i.e., 5th-2), caterpillars of the appropriate groups were given an immune challenge. One day after the immune challenge, (i.e., 5th-3) plasma was collected from all caterpillars and assayed as described below. At this time point (5th-3), starved caterpillars have been on the cellulose diet for 2 days. They have less lipid in their bodies, and less glucose, protein and trehalose in their hemolymph compared with caterpillars maintained on the normal diet, demonstrating that this diet significantly reduced resources ([19]).

### 2.3 Plasma melanization assay

Previously, we had shown that starvation increased phenoloxidase activity in *M. sexta* ([19]). However, we did not test whether this led to increased melanization. Melanization requires the availability of certain substrate molecules in the hemolymph ([28]), and these may not be readily available in starved caterpillars. To assess their melanization capacity, blood was collected from chilled caterpillars by snipping the right A6 leg with clean ice-cold scissors and allowing the blood to flow into an ice-cold centrifuge tube. A refrigerated centrifuge tube was pre-chilled to 2oC and the hemolymph was spun at 4000g for 12 min. The supernatant was removed carefully so as not to disturb the pellet containing the hemocytes. 70 µL of cell-free plasma was added to 20 µL of 1% Cetylpryidinium in a 96 well plate to measure the total melanization capacity of the plasma in vitro. Another 70 µL of cell-free plasma from the same tube was added to 20 µL of PBS to measure the spontaneous melanization rate of the plasma. The spontaneous melanization rate demonstrates how rapidly the plasma responds to foreign material (i.e., the plastic well plate) in vitro without an external activator (i.e., Cetylpryidinium). The plate was then run on a spectrophotometer at 450 nm for 10 minutes with readings taken at 30 s intervals. The Vmax (optical density milliunits/minute) was recorded over the linear portion of the reaction. Data collection from the plate reader was done blind.

### 2.4 Procedure for the metabolomics analysis

Plasma was collected as described above. Plasma was snap frozen with liquid nitrogen and then transferred to a -80C freezer prior to metabolomic and proteomic analysis. Metabolomic analysis was performed by the Metabolomics Innovation Center at the University of Alberta. There were 8 biological samples for each of the 4 treatments.

Full details of the non-targeted metabolomics procedure are provided in the Supplemental Material. In brief, metabolomics was performed using high performance chemical isotope labelling LC-MS. Proteins were removed from the sample using 45 µL of LC-MS grade methanol before chemical isotope labelling. Samples were divided into 4 aliquots. One aliquot was used for amine-/phenol-labelling, and one for carboxy-labelling. Prior to LC-MS analysis, quality control samples were prepared by an equal volume mix of a 12C-labelled and a 13C-labelled pooled sample.

The samples were analyzed using Rapid LC-MS analysis on the HP-CIL Metabolomics Platform on a Thermo Scientific Vanquish LC linked Bruker Impact II QTOF Mass Spectrometer and an Agilent eclipse plus reversed phase C18 Column (150x2.1mm, 1.8 µm particle size). All samples passed the quality control checks. Metabolites were identified by comparing the retention time and mass of sample components with CIL and LI libraries. All data were normalized prior to analysis.

#### 2.4.1 Data analysis for the metabolomics.

Initial analyses were performed by the Metabolomics Innovation Centre. See Supplementary Material for more details. To maintain statistical power, both the metabolomics and proteomics results were analyzed using pre-determined contrasts: control vs starved, control vs IC and IC vs ICSt. Metabolites were also analyzed and visualized using Qlucore Omics Explorer (Version 3.11.17). The q-value, a significance threshold that takes into account multiple tests reducing the false discovery rate, was set at <0.05. This threshold results in p-values below 0.05. We also used a 2 fold cut-off to focus on the most robust changes. Further details are in the Supplementary Material.

### 2.5 Procedure for the proteomics analysis

Proteomics and initial analysis was performed by the Biology Mass Spectrometry Centre at Dalhousie University. There were 10 biological samples/treatment. The following methods were provided by the facility.

Protein was extracted from 20 μL plasma by combining it with 80 μL of a solution of 1M ammonium bicarbonate, 4M urea, 5mM MgCl2, 0.1% (v/v) Triton X100, 0.01% (w/v) SDS, 5mM dithiothreitol, and 0.5X cOmplete protease inhibitor (Sigma). To support degradation of DNA/RNA, 2 μL of a nuclease solution (1U/μL benzonase) was added to the solution and incubated at 37°C for 10-minutes. After nuclease digestion, protein concentration of the resulting lysate was measured using an EZ-Q assay (Thermo Scientific). Protein cleanup prior to digestion was carried out using an adapted version of the previously described SP3 protocol (PMID: 25358341, 30464214). Specifically, 5 μL of a mixture of Sera-Mag carboxylate SpeedBeads (CAT#45152105050250 and CAT#65152105050250, prepared as 100 mg/mL stock solution in water) was added to 50 μg of protein for each sample prior to 4X volumes of acetone. Mixtures were incubated on a Thermomixer at 37°C for 5-minutes at 800 rpm prior to centrifugation at 5,000g for 5-minutes. After spinning, the supernatant was discarded and beads rinsed using 800 μL of an 80% (v/v) ethanol solution with pipetting to resuspend the beads. Mixtures were centrifuged at 5,000g for 5-minutes and the supernatant discarded prior to the addition of 1 μg of trypsin/rLysC mix (Promega) in 100 μL of digestion solution (100mM ammonium bicarbonate, 1mM CaCl2) and incubation at 37°C for 16-hours in a Thermomixer with mixing at 800 rpm. After digestion, peptides were centrifuged at 12,000g for 2-minutes and the supernatant recovered to a fresh tube containing 5 μL of a 10% (v/v) solution of trifluoroacetic acid. To desalt peptides prior to mass spectrometry (MS) analysis, an HPLC system (Agilent 1290 Infinity II with a DAD module) equipped with a reversed-phase column (CORTECS T3 1.6μm, 2.1x50mm, Waters) was utilized. Specifically, peptides were injected into a column equilibrated at 4% mobile phase B (0.1% formic acid in acetonitrile), rinsed for 1.5-minutes at a flow rate of 1mL/min, and subsequently eluted for 0.8-minutes at 80% mobile phase B (mobile phase A = 0.1% formic acid in water). Desalted peptides were dried in a SpeedVac centrifuge and reconstituted in 1% (v/v) formic acid in water at a concentration of 1 μg/μL based on the 214nm UV signal from the HPLC desalting step.

Peptide samples were analyzed using a data-independent acquisition (DIA) routine on an Orbitrap Fusion Lumos mass spectrometer (MS) (Thermo Scientific). Samples were introduced to the MS using a M-Class liquid chromatography (LC) instrument (Waters) equipped with a trapping-analytical column setup. For injection, peptides were initially trapped on a pre-column (ACQUITY UPLC M-Class Symmetry C18 Trap, 180µm x 2 cm, 5µm beads, Waters) and then separated by an analytical column (nanoEase M/Z Peptide BEH C18, 75µm x 25 cm, 1.7µm beads, Waters). Samples were initially trapped for 3-minutes at a flow rate of 20 µL/min of 1% mobile phase B (0.1% formic acid in acetonitrile). Gradient separation of trapped peptides started at an initial condition of 1% mobile phase B, ramped to 8% B in 1-minute, to 30% B in 25-minutes, to 80% B in 0.5-minutes, hold at 80% B for 2-minutes, ramp to 1% B in 0.5-minutes, and a final hold at 3% B for 11-minutes for a total run time of 40-minutes (mobile phase A = 0.1% formic acid in water, flow rate = 300nL/min). Each peptide sample was injected three individual times (40-minute acquisition time for each), with each acquisition covering analysis of a separate mass range (injection 1 = 430–550, 2 = 550–670, 3 = 670–910 m/z). Specifically, the Orbitrap Lumos MS was globally set to use a positive ion spray voltage of 2200 V, an ion transfer tube temperature of 275°C, a default charge state of 3, and an RF Lens setting of 45%. The complete duty cycle of the acquisition method consisted of two MS1 scans and two sets of windowed MS2 scans (order MS1 - MS2 - MS1 - MS2). For the first, low mass range injection, the initial MS1 scan covered a mass range of 415-565m/z at a resolution of 60,000 with an AGC target of 4e5 (100%) and a max injection time set to ‘Auto’. The following set of DIA MS2 scans covered a precursor range of 430-550m/z with an isolation window size of 4m/z (0m/z overlap) for a total of 30 scan events. Each scan used an HCD energy of 30% and covered a defined mass range of 200-1800m/z at a resolution of 30,000 with a normalized AGC target of 1000%, and the maximum injection time set to ‘Auto’. Loop control was set to ‘N’ with a value of 30 spectra. The next MS1 scan and following DIA MS2 used the same settings as the previous with the exception that the MS2 precursor mass range was set to 428-552m/z to give a 2m/z stagger with the previous scan windows. For the second, medium mass range injection, the scan settings were the same as for the low mass injection with the following changes: 1. MS1 scan range of 535-685m/z; 2. DIA-MS2 precursor scan ranges of 550-670m/z and 548-672m/z; 3. Loop control number of spectra = 30. For the third, high mass range injection, the scan settings were the same as the others with the following changes: 1. MS1 scan range of 655-925m/z; 2. DIA-MS2 precursor scan ranges of 670-910m/z and 666-914m/z; 3. DIA-MS2 isolation window of 8m/z; 4. Loop control number of spectra = 30. All scan data were acquired in centroid mode. DIA-MS raw data were processed using DIA-NN (version 2.0) (PMID: 31768060). Specifically, a representative *Manduca sexta* proteome fasta database (version 01/2025, 21,016 entries, includes contaminants) was provided to DIA-NN along with all of the raw data in order to perform an initial spectral library generation step (default settings for library generation, --min-pr-mz 430 --max-pr-mz 910 --min-pr-charge 2 --max-pr-charge 4, MBR disabled). Afterwards, a subset of raw files for each mass range were searched together against this generated spectral library using DIA-NN with ‘unrelated runs’ activated to derive appropriate mass error and scan window settings. The entire set of raw riles for each mass range were then searched against the spectral library using the derived mass error and window settings with MBR enabled. Resulting report files were passed to the iq package (Lib.Q.Value = 0.01, Lib.PG.Q.Value = 0.01, Q.Value = 0.01, PG.Q.Value = 0.05) in R to generate estimates of protein abundance (PMID: 31909781).

#### 2.5.1 Data analysis for proteomics.

Proteomics values were normalized for each protein on a log_2_ scale. Protein identifications that were based on less than 10% of the total protein sequence were omitted to focus on those identifications that were most robust. A GO analysis on differentially abundant proteins was run using PANTHER (https://pantherdb.org). The Benjamini-Hochberg false discovery rate was set to q < 0.05. Further analyses were done using Qlucore Omics Explorer. The false discovery rate was set at q < 0.05, with an additional cut-off of a minimum 2 fold change. See Supplementary Material for more details.

### 2.6 Nodulation assay to assess cell-mediated immunity

Methods were as previously described in Adamo et al. [48]. To examine the effect of the treatments on nodule formation, we collected Control (n = 37), IC (n = 37), St (n = 37), ICSt (n = 37), Wtm (Weight-matched controls, n = 36), and ICWtm (Immune challenged weight-matched controls, n = 42) caterpillars. All immune challenged caterpillars were given 40 µL of the heat-killed challenge described above one day prior to collection. A midline dorsal incision was used to open the body cavity of chilled caterpillars. The gut was removed and examined for nodules. The number of brown/black spots found in the body cavity between the second abdominal segment and terminal ganglion of the ventral nerve cord were also counted. Counts were done blind. A subset of animals were randomly chosen for a double count. The interobserver reliability was high (Spearman’s correlation, r = 0.935, p = 0.0001, n = 11 pairs).

Because cell-mediated immunity depends on plasma glucose levels in *Drosophila* ([1]), we measured the abundance of glucose and trehalose (an intermediate glucose storage molecule in insects, [49]) in Control, Starved, IC, and ICSt plasma (n = 6/group) using targeted mass spectrometry analysis. The analysis was conducted by the Biology Mass Spectrometry Centre at Dalhousie University (see Supplementary Material for methods).

### 2.7 Effect of starvation on defense against bacterial (*Bacillus cereus*) infection

We examined the ability of starved caterpillars to survive a bacterial challenge. We used the Gram positive bacterium *Bacillus cereus* (Microkwik culture from Carolina Biological, Burlington, NC, USA). This bacterium has been used previously with *M. sexta* (e.g., [19]). We used three groups of caterpillars: normal fed caterpillars, starved caterpillars and weight matched controls (Wtm) who were the same weight as the starved caterpillars, but approximately one day younger (5th instar day 1–2). We used the same treatment protocol as described previously. Starved caterpillars were given the non-nutritive cellulose diet on 5th-1. On 5th-2 control caterpillars were injected with the LD50 dose of live *B. cereus* (2 x 104 cells, [19]). The injection volume was adjusted for caterpillar weight for Starved and Wtm caterpillars by calculating the percentage of the Starved and Wtm caterpillar weight compared with the average control weight, and the dose was then adjusted by that percentage. Therefore, each caterpillar received approximately the same number of bacteria/mL hemolymph. The results from first time point (5 min, Fig 12a) of the experiment described below supports this assumption, with all animals showing approximately the same number of colony forming units (CFUs)/60 µL of hemolymph. The number of caterpillars that died were assessed for the next 2 days. We used a 48 h cut-off for assessing mortality because starved caterpillars become increasingly debilitated without nutrition ([50]). We wanted to reduce the possibility that mortality was being driven by the further loss of resources in starved caterpillars, and focus on the effect of the change in their immune system pathways.

To test our hypothesis that the pro-inflammatory state of starved caterpillars allows them to clear bacteria more rapidly early in infection, we examined the number of CFUs in the hemolymph at 5 min, 1h, 3 h and 24 h post infection. Methods are based on Stanley-Samuelson et al. [51]. Bacterial counts were made at only one time point per caterpillar. We used a similar procedure as described above (including controlling the dose for caterpillar weight), but increased the bacterial dose 5-fold. Caterpillar hemolymph was collected at 5 min, 1h, 3h or 24h after bacterial injection. 60 µL of hemolymph was diluted (1:4 autoclaved PBS) and plated on sterile nutrient agar plates in duplicates and the colonies were counted the next day. The average CFUs/plate was recorded. Plates from control caterpillars grew no bacterial colonies (n = 10). Because the differences in CFUs between the initial time point and the remaining time points were more than an order of magnitude in size, the data showed significant heteroscedasticity, even after log transformation. Therefore, we compared bacterial counts across groups at each time point.

To test whether starved caterpillars increased melanization in response to infection, we assessed the melanization rate of the hemolymph of caterpillars 5 minutes after injecting them with live *B. cereus*. Bacterial injections were done as described above. Melanization rate was assessed as described in section 2.3.

### 2.8 The effect of GSH on immune function in starved caterpillars

To test whether increasing the amount of GSH in the plasma would reduce spontaneous melanization, caterpillars from each treatment group (Control, Starved and Wtm) were randomly allocated to receive either 20 µL of 10mM GSH/2g caterpillar weight, or the same amount of PBS (Sigma, P4417) (i.e., 20 µL of PBS/2g caterpillar weight) on 5th day 2. Five min after caterpillars were injected with PBS or GSH, blood was collected and melanization rate was assessed as described previously.

To test the hypothesis using a different method, we added cysteine (50 g cellulose + 150 mL 10mM cysteine solution in double distilled water) to the non-nutritive cellulose diet for an additional group of caterpillars. The cysteine amounts were adapted from Kumar et al. [52]. We measured the abundance of cysteine and GSH in the plasma of caterpillars fed the cysteine fortified diet (n = 10/group) as well as in Controls and Starved caterpillars using a targeted mass spectrometry analysis. This analysis was conducted by the Biology Mass Spectrometry Centre at Dalhousie University (see Supplementary Material for methods). We then tested whether caterpillars fed cysteine-enriched cellulose showed a reduction in in vitro melanization using the methods described previously.

We also examined the effect on caterpillar survival of injecting GSH (20 uL of 10mM GSH/2g caterpillar weight), or the same volume of PBS, 5 min after injecting live *B. cereus* in Controls, Starved and Wtm caterpillars. The same bacterial concentration was used as described above for bacterial plating. Survival was examined over the next 2 days.

### 2.9 Whole organism effects: Defensive behaviour assay

Previously we had shown that an immune challenge reduces the force of the defensive strike, an important component of the animal’s defensive behaviour ([11]). This decline resulted in increased predation ([11]). We test if defensive behaviour also shows a change in its behavioural structure given the involvement of muscle in immune function ([7,11]).

We constructed a ‘Manduca tree’ to allow caterpillars time to acclimate on a horizontal wooden dowel (2 mm diameter, 12 cm in length) before testing, as used in other studies of *M. sexta* defensive behaviour (e.g., [53]). The wooden dowels were affixed to a vertical wooden rod (1 cm diameter, 25 cm tall) attached to wooden stand. Caterpillars were allowed to acclimate for 2 minutes. Using a von Frey hair (4.31 mN), the right A6 abdominal leg was stimulated every 10 sec for 30 trials, or until 3 consecutive stimulations did not produce a response. The defensive behaviours used after each tactile stimulation were recorded (i.e., Single defensive strike, Repeated defensive strikes, Partial defensive strike, Use of Mandibles, Retraction of the A6 leg, Climbing the vertical rod, and No response). There were 5 groups: Control (n = 33), Starved (n = 26), Immune Challenged (IC, n = 26)), ICSt (n = 21) and Wtm (n = 18). Behaviour was scored blind.

The behavioural data were analyzed using a multivariate analysis. Patterns across the data were studied using a principle components analysis (PCA). For the PCA, behavioural scores were normalized by converting them to z-scores.

### 2.10 Statistics

Data other than the metabolomics and proteomics results were analyzed using SPSS (v. 28 and 29, IBM) or Prism (v. 10 and 11, Graphpad). Outliers were removed in accordance to Hoaglin and Iglewicz [54]. All data were tested to ensure that they met the assumptions of the statistical test performed. When data were not normally distributed, non-parametric tests were used ([55]). Multiple statistical tests on the same data set were corrected using the Benjamini-Hochberg procedure ([56], q < 0.05).

## 3 Results

### 3.1 In vitro melanization was elevated in starved caterpillars

**Fig 3:**
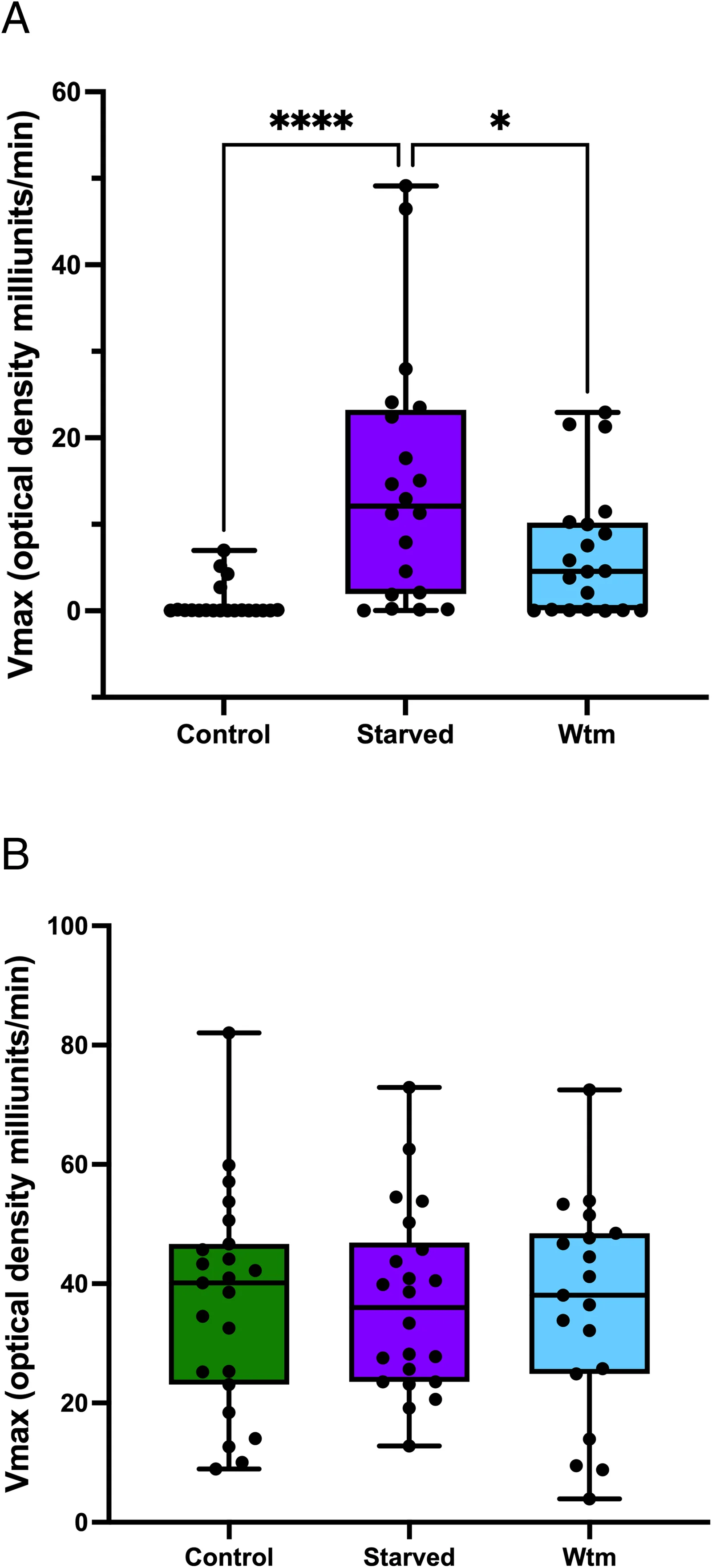
(a) Spontaneous melanization was higher in starved caterpillars. 1-way ANOVA (F(2, 57)=10.53, p < 0.0001. The central bar line denotes the median, with the top of the bar representing the 75th percentile, and the bottom of the bar representing the 25th percentile. Error bars show the maximum and minimum values. N = 20 for all groups. Asterisks denote significant differences across groups. Dots represent individual data points. *p < 0.05,* p < 0.0001. Wtm – weight matched control. (b) Total melanization capacity of the plasma. There were no significant differences across the 3 groups (1 way ANOVA (F(2, 61)=0.011, p = 0.99). The central bar line denotes the median, with the top of the bar representing the 75th percentile, and the bottom of the bar representing the 25th percentile. Error bars show the maximum and minimum values. Dots represent individual data points. Control n = 23, Starved n = 22, Wtm (weight matched control) n = 19.

Spontaneous melanization occurred at a faster rate in starved caterpillars than in controls (Fig 3a, 1 way ANOVA (F(2, 57)=10.53, p < 0.0001; Dunnett’s multiple comparisons test, p < 0.0001), or in Wtm caterpillars (Dunnett’s multiple comparisons test multiple comparisons test, p = 0.02; n = 20/group). There was no difference in spontaneous melanization rate between control and Wtm caterpillars (Dunnett’s multiple comparisons, p = 0.10). The total in vitro melanization capacity of the hemolymph did not differ across groups (Fig 3b, 1 way ANOVA (F(2, 61)=0.011, p = 0.99).

### 3.2 Plasma metabolomics

**Fig 4:**
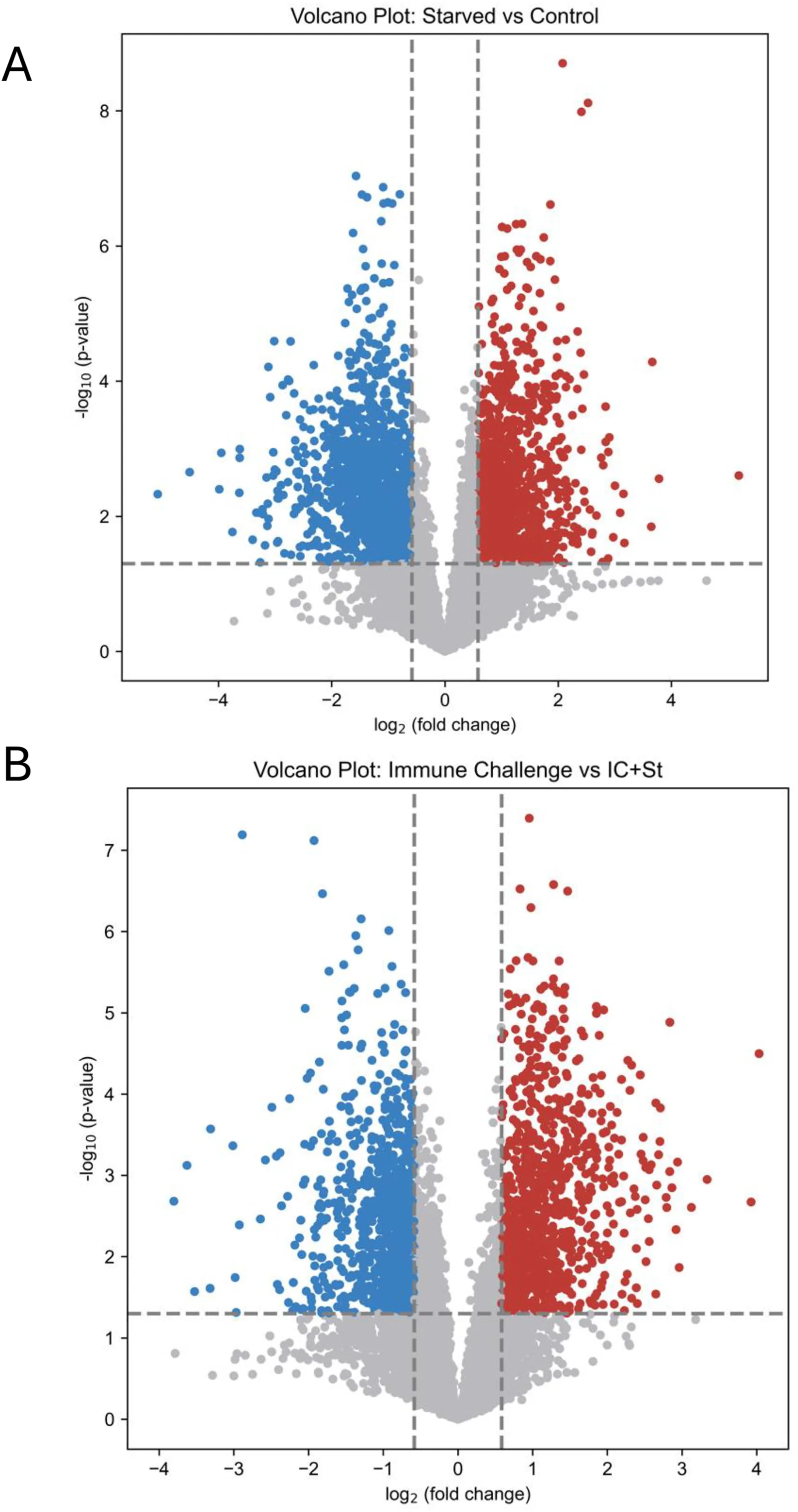
Volcano plots. Volcano plots were constructed by plotting the fold change of each metabolite against its p-value. Fold changes > 1.5 (red dots) or < 0.67 (blue dots). Both identified and unidentified metabolites are included in this plot. A Starved relative to Control caterpillars (N = 8/group). 1215 metabolites increased in starved caterpillars relative to control (red dots) and 1288 metabolites decreased (blue dots). B Immune Challenge and Starved (ICSt) relative to immune challenged (IC) caterpillars (N = 8/group). 1069 metabolites increased in ICSt vs IC (red dots) and 963 metabolites decreased (blue dots).

Volcano plots showed that starvation (St, n = 8) produced a mix of increased and decreased abundance of metabolites relative to controls (Con, n = 8) (Fig 4a). Similarly, an immune challenge in starved caterpillars (ICSt, n = 8) both increased and decreased metabolites relative to immune-challenged fed caterpillars (IC, n = 8) (Fig 4b). In other words, metabolites did not universally decline at this level of starvation, but approximately the same number of metabolites increased as decreased in abundance (Fig 4a, 4b). Amino acid abundance in the plasma was not significantly different between starved and control caterpillars (Table 1 in S1 Text). However, there were changes in other metabolites that corroborate the earlier study ([19]) suggesting a decline in energetic resources after 2 days of starvation. For example, the increase in dipeptides in the plasma of starved caterpillars suggests increased protein breakdown (Tables 1 and 2 in S1 Text).

**Fig 5:**
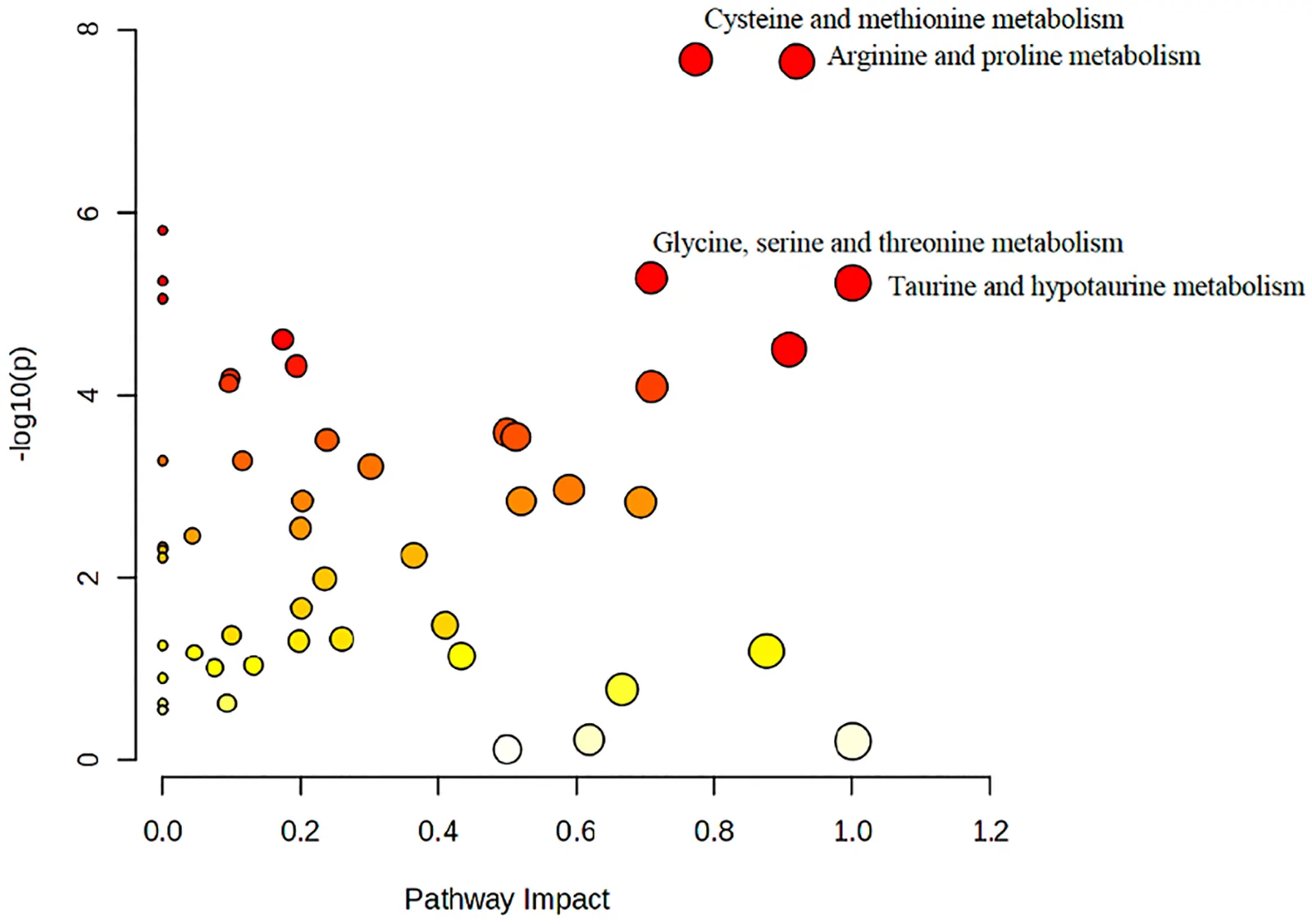
Metabolic pathway analysis showing the impact of ICSt on metabolic pathways relative to IC. Comparison between IC and ICSt was based on KEGG pathways for *Bombyx mori* (silkworm). Pathway Impact is a quantitative measure indicating the centrality of a metabolite within a network organization, and is visualized using circle size and color. Larger circles denote greater pathway impact, while a redder color signifies increased significance of the pathway. Pathways showing the most extreme effects are identified on the graph. ICSt = immune challenged and starved. IC = Immune challenged. N = 8/group.

The pathway analysis for metabolites that differed in abundance between ICSt and IC is shown in Fig 5 and summarized in Table 1. Table 1 shows that the majority of pathways significantly impacted by starvation (i.e., St and ICSt) play a role in preventing oxidative stress when found in the extracellular space (e.g., GSH, [57]). One exception was the arginine pathway that can promote the production of immune processes (e.g., NO) in the extracellular environment in *M. sexta* ([59]). It is noteworthy that although our heat-killed challenge induces a robust immune response in well-fed caterpillars (e.g., [19]), there was little evidence of significant metabolic pathway alterations in the plasma of immune-challenged caterpillars compared with controls. However, there were significant changes in individual metabolites (Fig S1 in S1 Text). Table 3 in S1 Text contains pathway analysis results for Control vs Starved, Control vs IC (immune challenged), and IC vs ICSt (immune challenged and starved).

**Table 1:** Metabolomic pathways significantly altered in the plasma of starved (St) and IC relative to controls and ICSt relative to IC.

Starved vs Control	IC vs Control	ICSt vs IC
Arginine+Proline	No Significant Effects	Arginine + Proline
Cysteine+Methionine		Cysteine + Methionine
Glutathione		Glutathione
Glycine, Serine, + Threonine		Glycine, Serine + Threonine
Taurine + Hypotaurine		Taurine + Hypotaurine
Beta-Alanine		Beta-Alanine
Tryptophan		Tryptophan
Alanine, Aspartate and Glutamate		Alanine, Aspartate and Glutamate

Orange pathways are frequently involved in protecting against oxidative stress in the extracellular space; Purple pathways can produce molecules involved in immunity and oxidative stress (e.g., NO). See [57,58].

IC = immune challenged, ICSt = immune challenged and starved.

**Fig 6:**
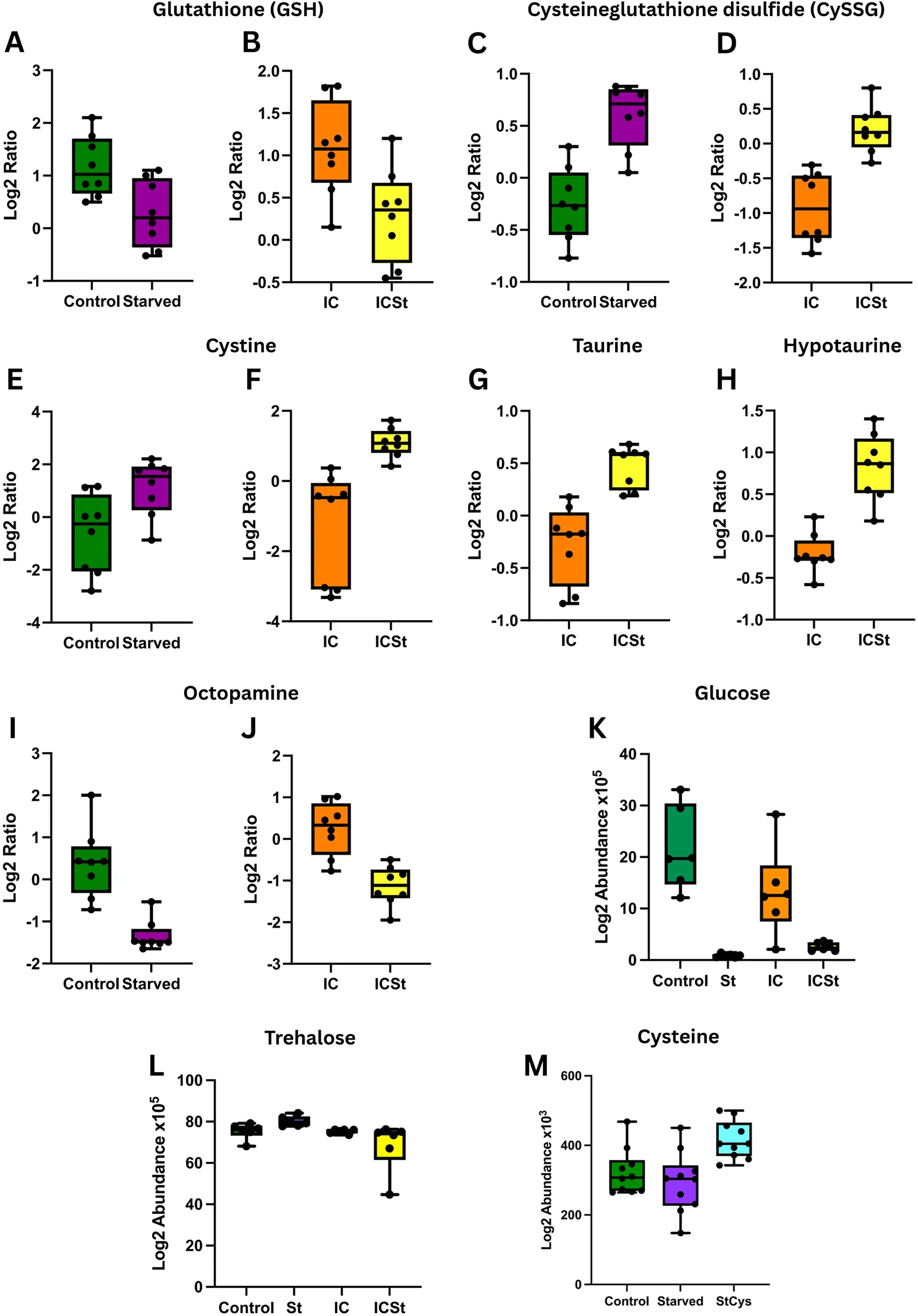
Box plots showing significant differences in the abundance of metabolites in the plasma. The metabolite is identified above the corresponding boxplot(s). The x-axis identifies the comparison across groups. A) Glutathione (GSH) in Starved relative to Control caterpillars. B) GSH in ICSt relative to IC caterpillars. C) Cysteineglutathione disulfide (CySSG) in Starved relative to Controls. D) CySSG in ICSt relative to IC. E) Cystine in Starved relative to Control caterpillars. F) Cystine in ICSt relative to IC. G) Taurine and H) Hypotaurine in ICSt relative to IC. I) Octopamine in Starved relative to Control caterpillars. J) Octopamine in ICSt relative to IC. In plots A-J, the y-axis represents the log_2_ ratio of the peak intensity (i.e., 12C-samples/13C-reference) for the corresponding metabolite. N = 8/group. K) Glucose abundance in Control, St, IC and ICSt plasma. See text for statistical analysis. L) Trehalose abundance in Control, St, IC and ICSt plasma. No significant differences across groups. M) Cysteine abundance in Control, Starved and StCys plasma. Statistical tests given in text. In plots K-M, the y-axis denotes the log_2_ abundance relative to standards for the corresponding metabolite, n = 6. In plot M, n = 10/group. For all plots, the central line represents the median, and the top and bottom of the bar represents the 25th and 75th percentiles. The error bars denote minimum and maximum values. p < 0.05. Green bars denote Controls and purple bars denote Starved (St) caterpillars. Orange bars denote IC (immune challenged) and yellow bars denote ICSt (immune challenged and starved). Dots represent individual samples.

The powerful antioxidant GSH showed a decrease in abundance in starved caterpillars, including ICSt (Fig 6A + B). There was a concomitant increase in Cysteineglutathione disulfide (CySSG), an oxidized form of GSH ([60]) (Fig 6C + D), as well as an increase in cystine, the oxidized form of cysteine (Fig 6E + F). This metabolite is a sign of inflammation ([61]). GSH interacts with cystine, leading to the production of cysteine disulfide (CySSG, [62]). The increase in CySSG, coupled with declines in GSH, is also indicative of an inflammatory state ([61]). Unfortunately we were unable to measure glutathione disulfide, the usual oxidized from of GSH. These results suggest a decline in the reducing capacity of the hemolymph in starved and ICSt caterpillars. At the same time, we observed increased abundance of taurine (Fig 6G) and hypotaurine (Fig 6H) in ICSt relative to IC. These molecules have antioxidant properties, although they are less powerful antioxidants than GSH ([52,63]). There was no difference in taurine and hypotaurine abundance between starved and control caterpillars, or between IC and control caterpillars (S1 Table, and Fig 1 in S1 Text). Therefore the increase of these alternate cysteine-containing antioxidants is only observed in the dual challenge.

ICSt caterpillars showed an increase in abundance in dipeptides in the plasma relative to IC (Table 2 in S1 Text). This increase is probably a sign of increased proteolysis ([64]), but can also be a response to oxidative stress. Some dipeptides, such as tryptophyl-tyrosine (higher in ICSt relative to IC, S2 Table) can act as antioxidants ([65]). Starved caterpillars also showed an increased abundance of dopaquinone relative to controls, and a decline in cysteinyldopa (Fig 1), suggesting increased activity of the eumelanin pathway even without pathogen presence.

Octopamine is an important stress neurohormone ([66,67]). Its level in plasma declined significantly after 2 days of starvation (i.e., cellulose diet) (Fig 6I) relative to controls. OA showed no change in abundance in IC caterpillars relative to controls (Fig S1 in S1 Text). However, octopamine was lower in ICSt caterpillars compared with IC caterpillars (Fig 6J).

### 3.3 Plasma proteomics

**Fig 7:**
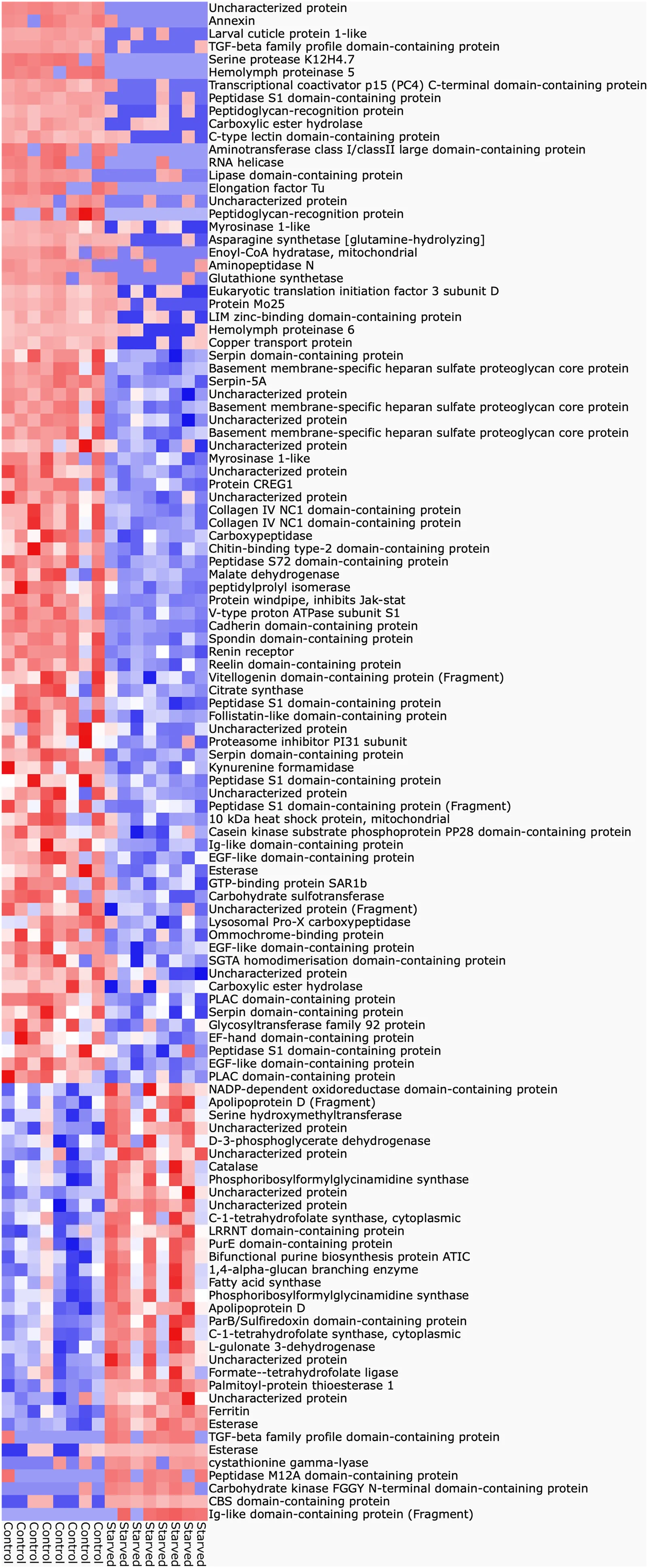
Heat map of the proteins that were significantly different (i.e., at least a 2 fold change, q < 0.05) in abundance in the plasma of starved caterpillars relative to Controls. Red squares demonstrate higher abundance, while blue squares represent lower abundance. The darkness of the colour reflects the amplitude of the increase or decrease. Scale color bar is shown in Fig S6 in S1 Text. n = 10/group.

**Fig 8:**
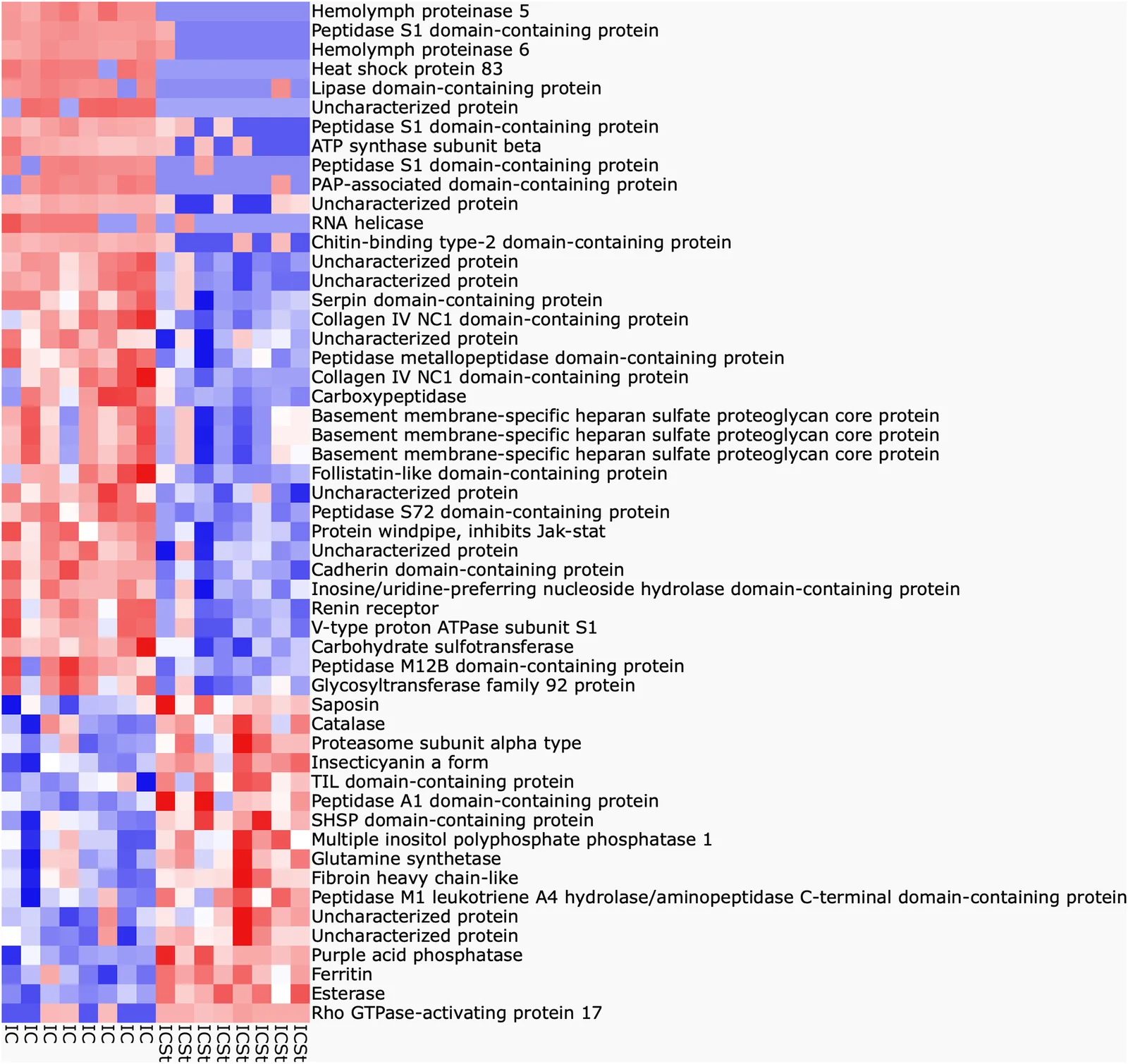
Heat map of the proteins that were significantly different (i.e., at least a 2 fold change, q < 0.05) in abundance in the plasma of ICSt caterpillars relative to IC caterpillars. Red squares demonstrate higher abundance, while blue squares represent lower abundance. The darkness of the colour reflects the amplitude of the increase or decrease. Scale color bar is shown in Fig S6 in S1 Text. n = 10/group. IC = immune challenged. ICSt = immune challenged and starved.

A proteomics assay of the plasma identified 1435 proteins that passed criteria (i.e., at least 10% peptide coverage), (S4 Table). We found 110 proteins that differed in abundance in the plasma of starved caterpillars relative to that of controls (Fig 7). The GO analysis found changes in serine related processes (Fig 2 in S1 Text). Fig 8 illustrates the 53 proteins that differed in abundance between IC and ICSt. A GO analysis of these proteins found that proteins involved in antioxidant activity and metabolic processes were significantly altered in abundance (Molecular Functions Fig S3 in S1 Text; Biological Processes, Fig S4 in S1 Text). Changes in the abundance in antioxidant proteins is summarized in Table 2.

**Table 2:** Changes in the abundance of proteins that can act as antioxidants.

Protein	IC Relative to Control	St Relative to Control	IC + St Relative to IC
Catalase	**ND**	**UP**	**UP**
Ferritin	**ND**	**UP**	**UP**
Thioredoxin	**Down**	**ND**	**UP**
Superoxide dismutase (SOD)	**Down**	UP [Cu/Zn]*	**UP**

ND – No significant difference.

*Starved caterpillars showed an increase in abundance in SOD [Cu-Zn] but not of other SOD subtypes.

IC = immune challenged, ICSt = immune challenged and starved, St = starved.

**Fig 9:**
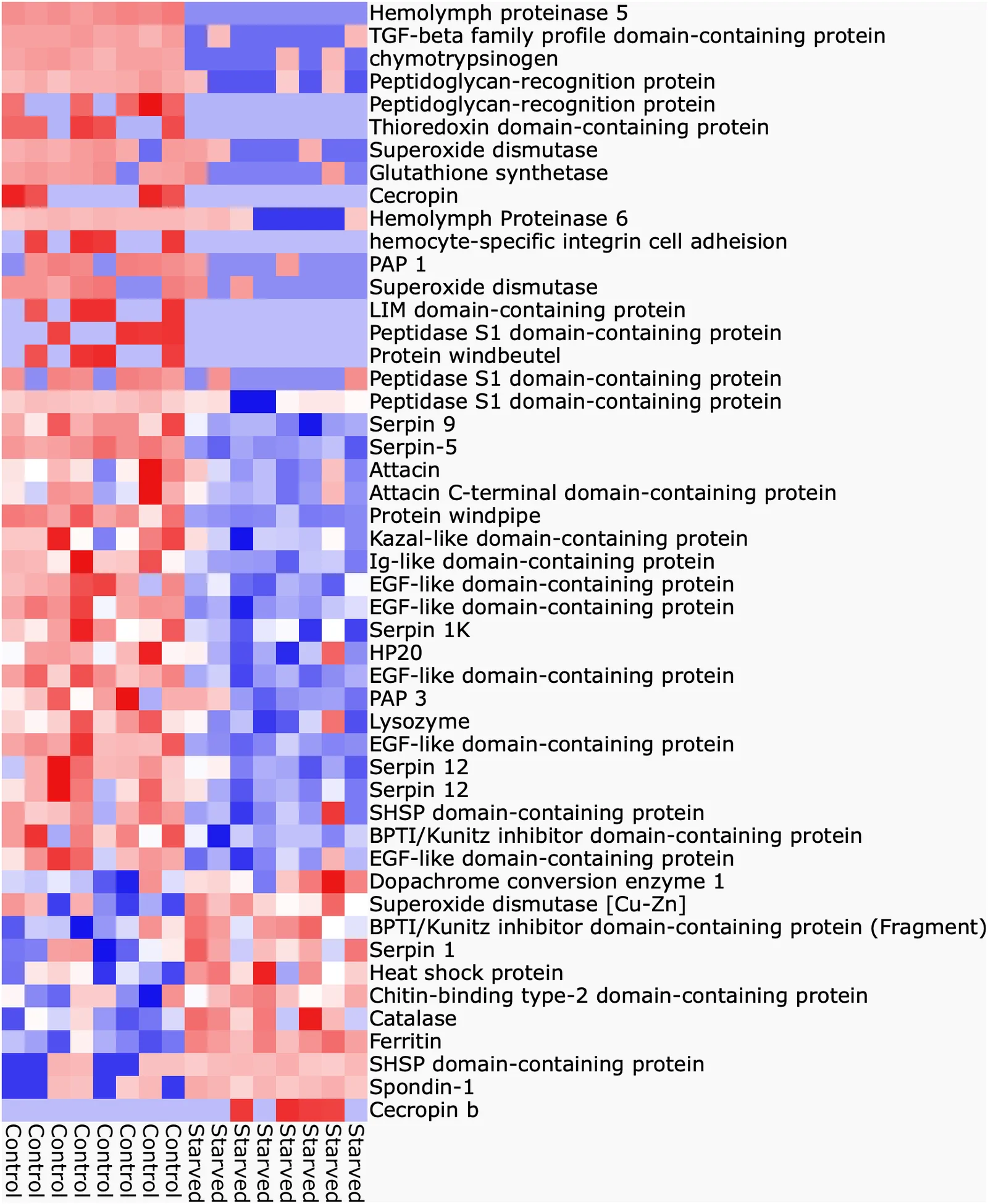
Heat map of the immune-associated proteins that were significantly different (i.e., at least a 2 fold change, q < 0.05) in abundance in the plasma of starved caterpillars relative to Controls. Red squares demonstrate higher abundance, while blue squares represent lower abundance. The darkness of the colour reflects the amplitude of the increase or decrease. Scale color bar is shown in Fig S6 in S1 Text. N = 10/group.

**Fig 10:**
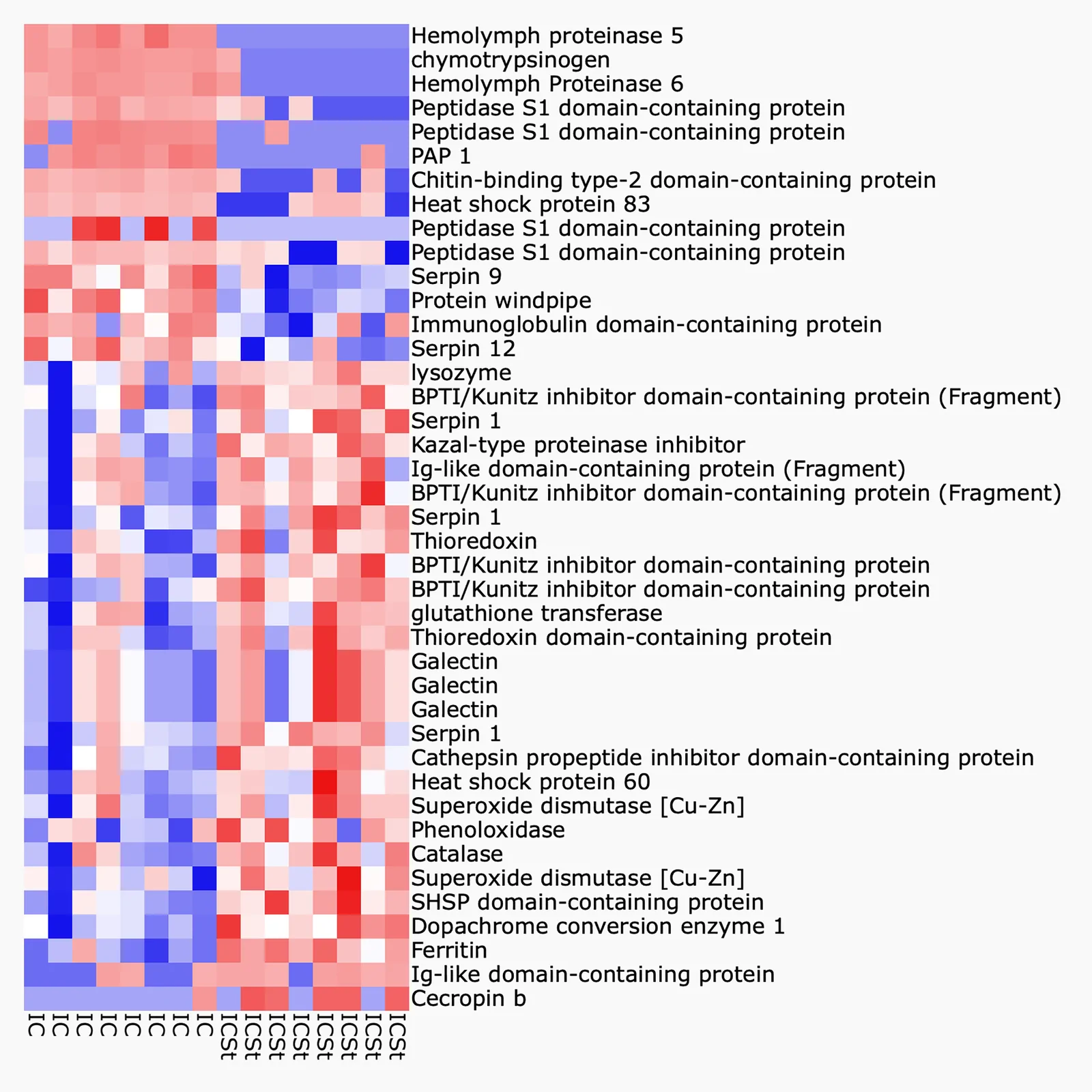
Heat map of the immune-associated proteins that are significantly different (i.e., at least a 2 fold change, q < 0.05) in abundance in the plasma of ICSt caterpillars relative to IC caterpillars. Red squares demonstrate higher abundance, while blue squares represent lower abundance. The darkness of the colour reflects the amplitude of the increase or decrease. Scale color bar is shown in Fig S6 in S1 Text. N = 10/group. IC = immune challenged. ICSt = immune challenged and starved.

We also observed a decline in the abundance of lysozyme and the antimicrobial peptide (AMP) attacin in starved caterpillars relative to controls. (Figs 7, 9). Nevertheless during an immune challenge, starved caterpillars (ICSt) were able to make AMPs. Some AMPs (e.g., cecropin b, galectin) had greater abundance in ICSt than IC (i.e., fed and immune-challenged) caterpillars (Fig 10).

**Fig 11:**
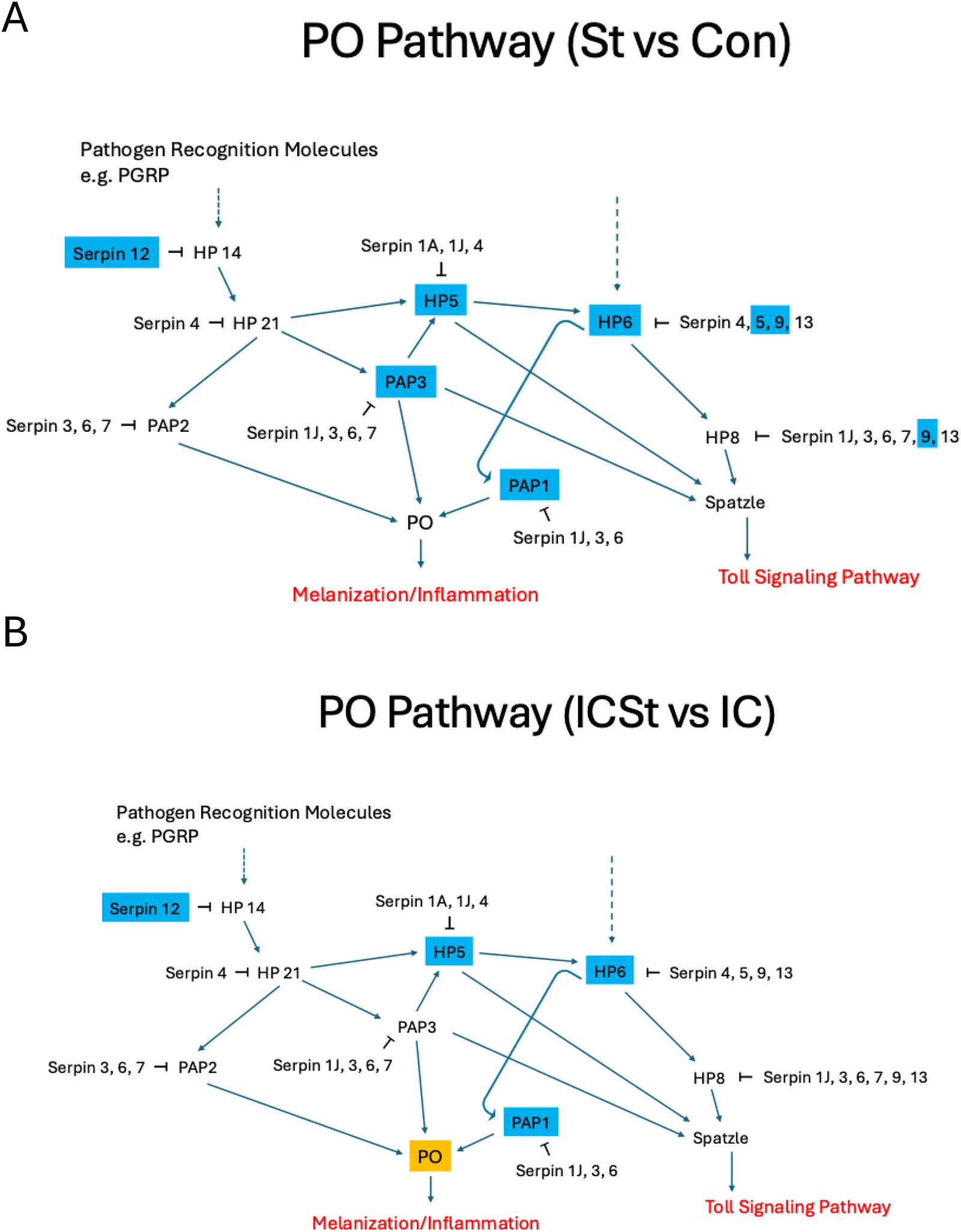
Simplified schematic of the PO pathway in *M. sexta* demonstrating changes in abundance in: a) Starvation (St) relative to Control (Con) and b) ICSt relative to IC. Blue rectangles denote proteins that were in lower abundance. Orange rectangles denote proteins that were increased in abundance. The magnitude of the relative changes is visible in Fig 10. Pathway adapted from [6,27,42]. IC = Immune-challenged, ICSt = Immune-challenged and Starved.

Using a combination of a GO analysis and Uniprot (uniprot.org) to identify immune proteins, we focused on the differences in abundance within this group of proteins (Figs 9, 10, 11, S5 Table). However, in some cases we could not make an exact identification. For example, Serpin-1 was increased in abundance in ICSt vs IC (Fig 10). However, there are several sub-types ([68]), and we were unable to determine which subtypes had changed. Because both the metabolomics and proteomics analyses showed changes in abundance of molecules in this pathway, we combined the information from both analyses. In the Discussion (Section 4.1) we describe how starvation, immune challenge, and a combined challenge impacted this important immune pathway.

Despite declining resources, Starved and ICSt caterpillars showed an increased abundance of proteins that assist with defending against oxidative stress. There appears to be a shift away from the use of glutathione as a major antioxidant, to enzymatic antioxidants (e.g., catalase, ferritin, thioredoxin, and superoxide dismutase (SOD) (Table 2).

### 3.4 Effect of starvation on defense against bacterial (*Bacillus cereus*) infection

There was no significant difference in mortality between fed, starved, and weight-matched caterpillars 48 h after bacterial (*Bacillus cereus*) infection [Chi-square (2 df)=0.08, p = 0.96, fed 36/44 alive, starved 33/44 alive, Wtm 32/40], despite the increasing resource deficit in the starved caterpillars at this time point (i.e., 3 days without nutrition).

**Fig 12:**
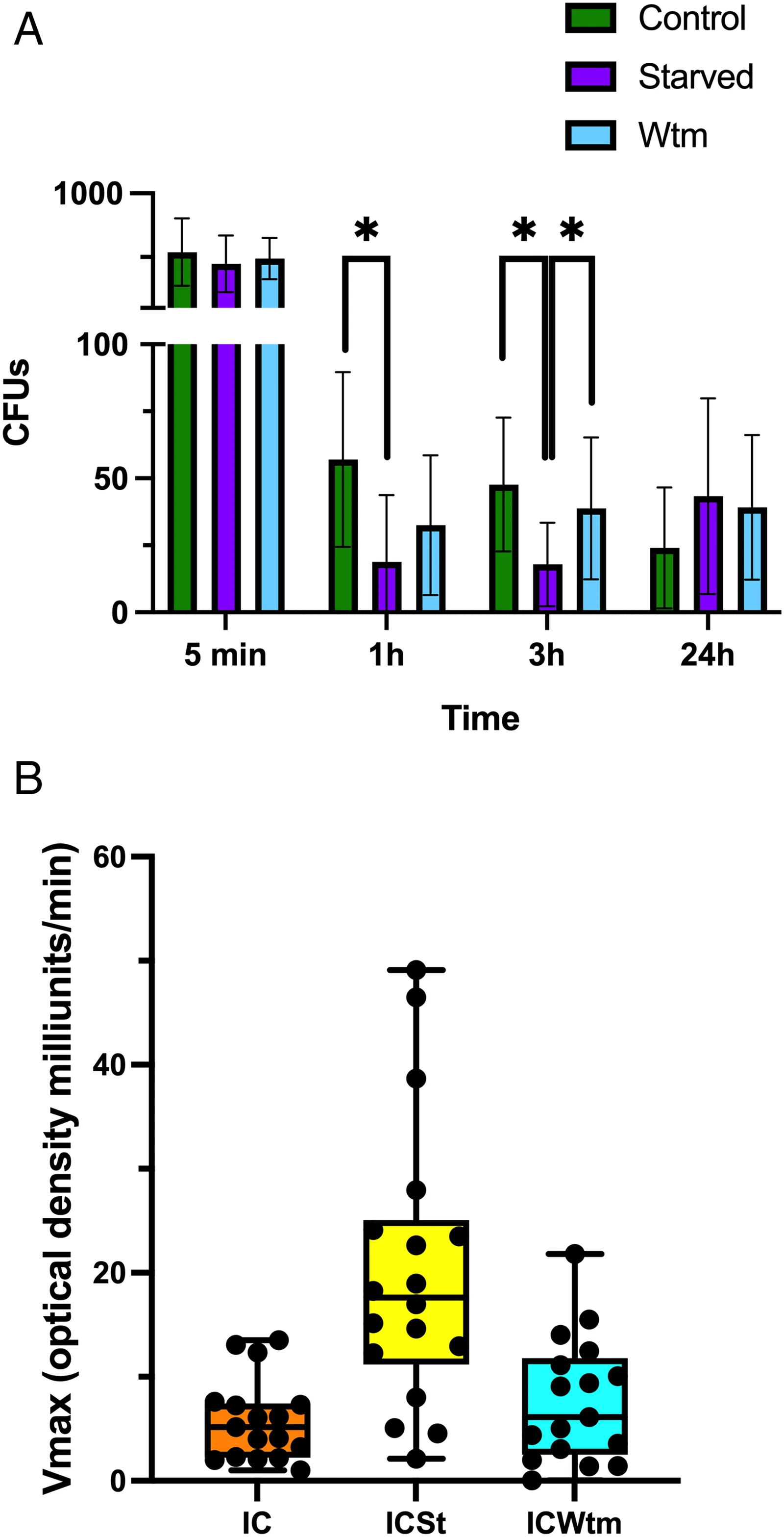
(a) Starved caterpillars showed fewer CFUs after bacterial infection than did control caterpillars or weight-matched caterpillars at 3h post-injection. Bars denote the average and the error bars show standard deviation. An asterisk denotes a significant difference q < 0.05. Note the interrupted y-axis and change in scale between the top and bottom parts of the graph. (b) Melanization rate is higher in starved caterpillars after a live bacterial challenge than in infected controls or weight-matched controls. The middle line in the box represents the median, with the bottom and top of the box representing the 1st and 3rd quartile. The error bars represent maximum and minimum values. Dots represent individual data points. IC. n = 17; ICSt, n = 18; ICWtm, n = 17. IC – controls injected with bacteria, ICSt- starved caterpillars injected with bacteria, ICWtm – weight-matched controls injected with bacteria.

CFU (colony forming units) were not significantly different across groups at the first time point (Fig 12a, 5 min, F(2, 42)=0.65, p = 0.53). However, starved caterpillars had fewer CFUs at 1 h and 3 h post injection than fed caterpillars, although this difference was not significant at 24 h (Fig 12a, 1 h (F (2, 43)=7.25, p = 0.0019, Tukey’s multiple comparison, control (n = 13) vs starved (n = 18), p = 0.0013, starved (n = 18) vs wtm (n = 15), p = 0.35; 3h F(2,44)=6.94, p = 0.0024, control (n = 15) v starved (n = 17), p = 0.0016,; 24 h (F2,42)=1.8, p = 0.177). Starved had significantly fewer CFUs at 3 h than weight-matched controls (Tukey’s multiple comparison, starved vs wtm (n = 15) p = 0.030, Benjamini-Hochberg correction, q = 0.033).

Melanization rate was higher in starved caterpillars after infection (n = 18) than it was in infected controls (n = 17) or weight-matched controls (n = 17). (Fig 12b. F(2, 49)=13.22, p < 0.0001; Tukey’s multiple comparison test, ICSt vs IC, p < 0.0001, ICSt vs ICWtm, p = 0.0004, IC vs ICWtm, p = 0.82).

### 3.5 Starved animals make fewer nodules than controls in response to an immune challenge

**Fig 13:**
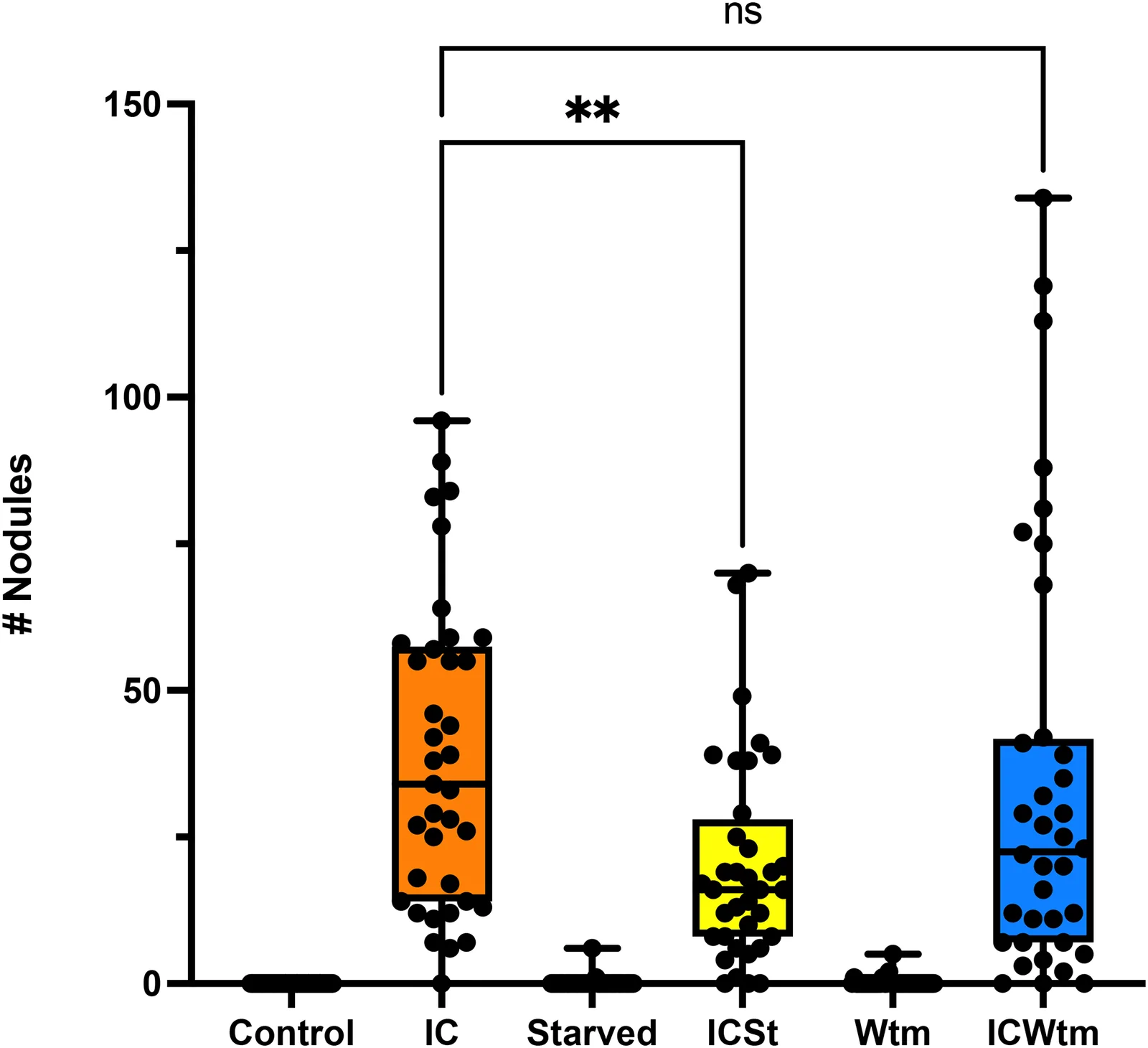
Number of nodules differs across groups. The central bar line denotes the median, with the top of the bar representing the 75th percentile, and the bottom of the bar representing the 25th percentile. Error bars show the maximum and minimum values. Dots represent individual data points. p = 0.01, ns – no significant difference. IC = immune challenged. ICSt = immune challenged and starved. Wtm = weight-matched control. ICWtm = Immune-challenged weight matched control.

Nodulation data from control animals was not normally distributed, because all of the values for control caterpillars were 0 (n = 37). Using a non-parametric analysis ([55]), we found that immune challenged caterpillars (i.e., IC (n = 37), ICSt, n = 36, and ICWtm n = 36) had more nodules than non-challenged (Control) caterpillars (Control (n = 37), St (n = 35), Wtm (n = 42), z = 4.6, p < 0.0001, Fig 13). Analyzing the immune challenged groups separately, we found that starved caterpillars had significantly fewer nodules than controls (Fig 13, 1 way ANOVA, F(2, 106)=4.45, p = 0.014; Dunnett’s multiple comparisons test, IC > ICSt, p = 0.01). However Wtm caterpillars were not significantly different from control (Dunnett’s multiple comparisons test, p = 0.72).

Plasma glucose abundance was significantly reduced in starved caterpillars (St and ICSt) relative to controls (Fig 6K, 1way ANOVA, F(3,20)=16.17, p < 0.0001, n = 6/group; Dunnett’s multiple comparisons, p < 0.0001). There was no significant difference in glucose abundance between Control and IC plasma (Dunnett’s multiple comparisons>0.05). Although glucose levels were lower in ICSt than in Control plasma, glucose levels in ICSt plasma were greater than that found in St plasma, p = 0.0025 (Benjamini-Hochberg correction, q < 0.05). There were differences in trehalose values across groups (n = 6/group, Fig 6L), but no significant pairwise comparisons (One way ANOVA, F(3, 20)=3.28, p = 0.04; Dunnett’s multiple comparisons test, p > 0.05).

### 3.6 The effect of GSH on immune function in starved caterpillars

**Fig 14:**
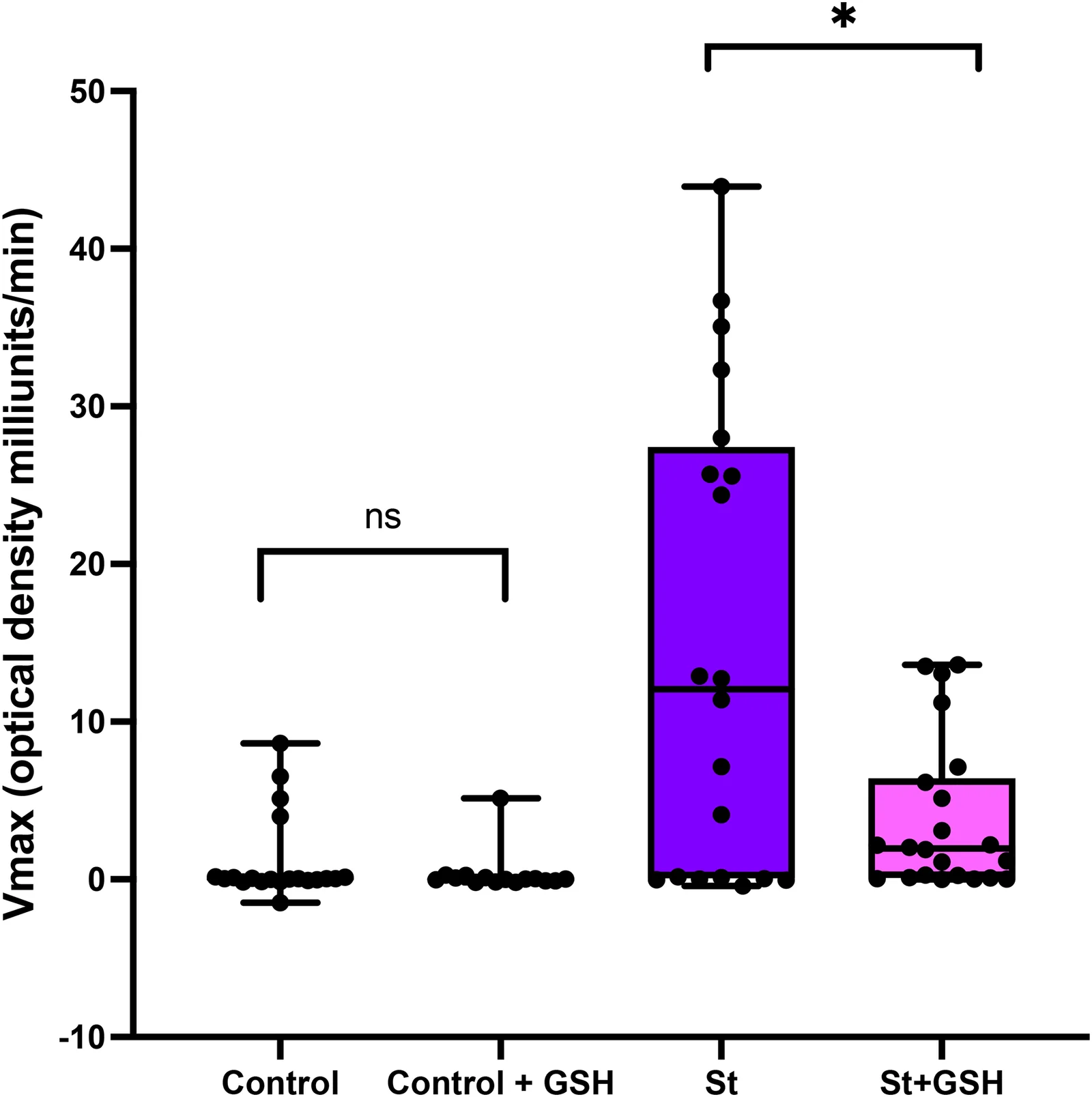
GSH reduced spontaneous melanization in St (starved) caterpillars. GSH had no significant effect on spontaneous melanization in control caterpillars. The central bar line denotes the median, with the top of the bar representing the 75th percentile, and the bottom of the bar representing the 25th percentile. Error bars show the maximum and minimum values. Dots represent individual data points. p < 0.0001, ns – no significant difference.

Injections of GSH reduced spontaneous melanization in starved caterpillars (Fig 14, Two-way ANOVA, Significant effect of GSH on Starved Caterpillars, F(1, 73)=19.4, p < 0.0001; Sidak’s multiple comparisons test, p < 0.0001, GSH-injected n = 22, PBS-injected, n = 20). However, GSH had no significant effect on fed caterpillars (Sidak’s multiple comparisons test, p = 0.95, PBS-injected n = 15, GSH-injected, n = 20), as expected given that fed caterpillars did not exhibit significant spontaneous melanization (Fig 3a). Starved caterpillars had higher melanization rate than did controls (F(1, 73)=9.25, p = 0.003) as found before, and there was a significant interaction effect (F(1,73)=6.94, p = 0.01).

**Fig 15:**
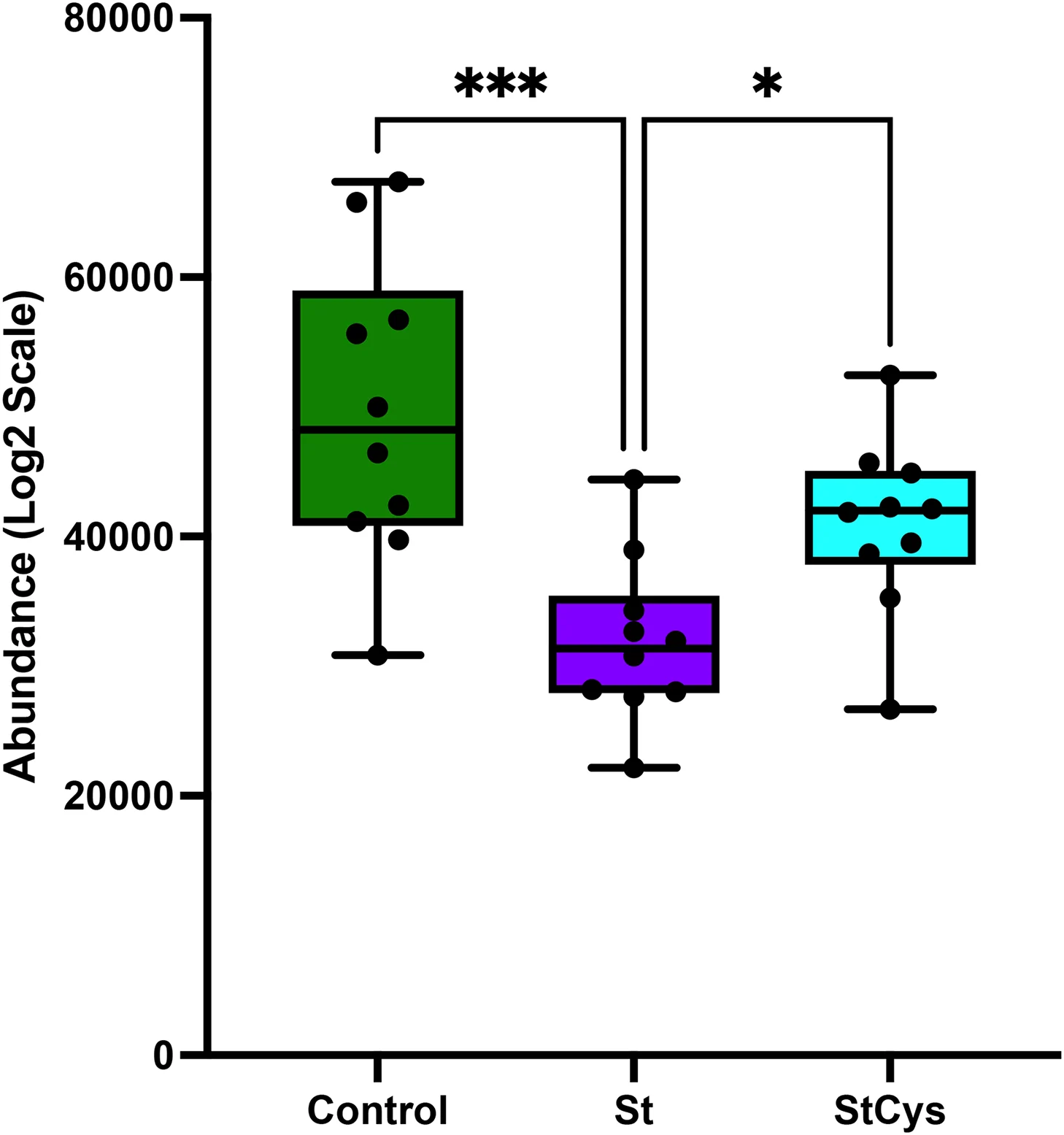
Fortifying the cellulose diet with cysteine increased GSH abundance in the plasma compared with starved caterpillars fed the basic cellulose diet. The central bar line denotes the median, with the top of the bar representing the 75th percentile, and the bottom of the bar representing the 25th percentile. Error bars show the maximum and minimum values. Dots represent individual data points. N = 10/group. StCys – caterpillars fed the cellulose diet fortified with cysteine. *p < 0.05, ***p < 0.001.

**Fig 16:**
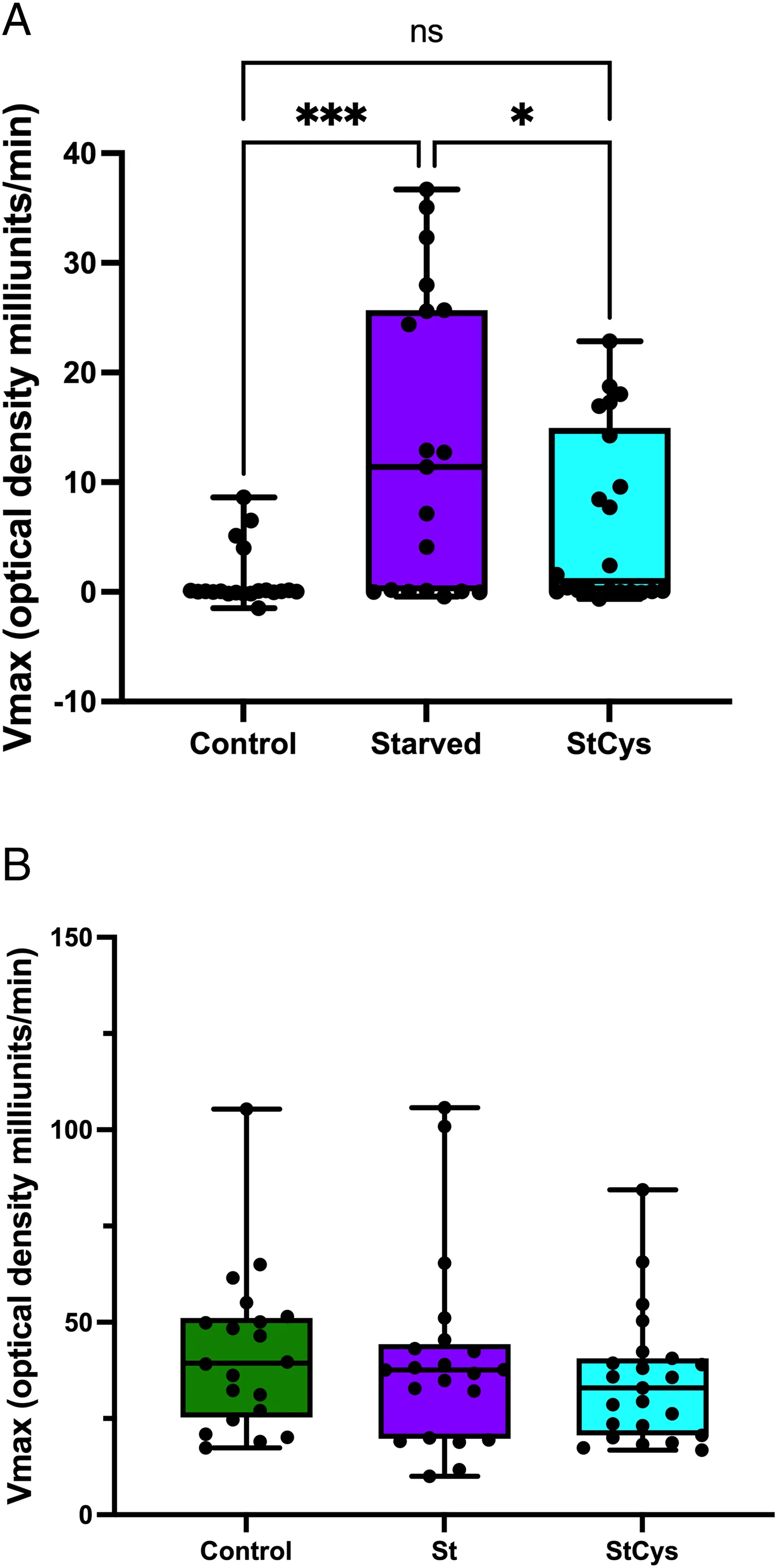
(a) Caterpillars fed cysteine-fortified cellulose showed significantly reduced spontaneous melanization. The central bar line denotes the median, with the top of the bar representing the 75th percentile, with the bottom representing the 25th percentile. Error bars show the maximum and minimum values. Dots represent individual data points. Control n = 20, St n = 19, StCys n = 22*.* *p < 0.05, ***p < 0.001. ns – no significant difference. St = starved (i.e., cellulose diet). StCys – caterpillars fed the cellulose diet fortified with cysteine. (b) Total melanization capacity was not significantly different across groups. The central bar line denotes the median, with the top of the bar representing the 75th percentile, with the bottom representing the 25th percentile. Error bars show the maximum and minimum values. Dots represent individual data points. Control n = 20, St n = 21, StCys n = 23. StCys – caterpillars fed the cellulose diet fortified with cysteine. St – Starved.

To corroborate our results using a different method, we ran an additional group of caterpillars that were given cellulose fortified with cysteine (n = 10/group). Two days after receiving the cysteine fortified cellulose diet, cysteine supplemented caterpillars (StCys) had more cysteine in their plasma than Starved. (Fig 6M, 1way ANOVA, F(2, 27)=8.31, p = 0.0015. Tukey’s multiple comparisons test. Starved vs StCys, p = 0.0016). Cysteine-fed caterpillars also had increased amounts of GSH in their plasma, compared to those that were fed cellulose alone (Fig 15, 1 way ANOVA (F (2, 27)=10.5, p = 0.0004, Dunnett’s multiple comparison test, Control > Starved, p = 0.0002, Cysteine-fed caterpillars> Starved, p = 0.03, n = 10/group). Caterpillars fed cysteine-fortified cellulose (n = 22) also had lower spontaneous melanization than did caterpillars fed cellulose alone (i.e., Starved, n = 17) (Fig 18a, F(2, 58)=8.85, p = 0.0004; Tukey’s multiple comparison test, Starved > StCys, p = 0.04, control n = 20). As found previously, control caterpillars had significantly less spontaneous melanization than did Starved (Tukey’s multiple comparison’s test, p = 0.0003. There was no significant difference in total melanization capacity across the 3 groups (Fig 16b, 1 way ANOVA, F(2, 61)=0.69, p = 0.51).

In starved caterpillars, injecting GSH 5 min after injecting *B. cereus* decreased survival 24 h later compared with PBS-injection (Fisher’s exact test. p = 0.04, n = 4/32 alive (GSH-injected), n = 12/34 alive (PBS-injected). However, there was no evidence that the GSH injection reduced survival in fed caterpillars (Fisher’s exact test, p = 0.79, GSH-injected 12/32 alive; PBS-injected 10/32 alive) or in Wtm controls (Fisher’s exact test, p = 0.9; GSH-injected 9/34 alive, PBS-injected 10/34 alive).

### 3.7 Whole organism effects: Defensive behaviour

**Fig 17:**
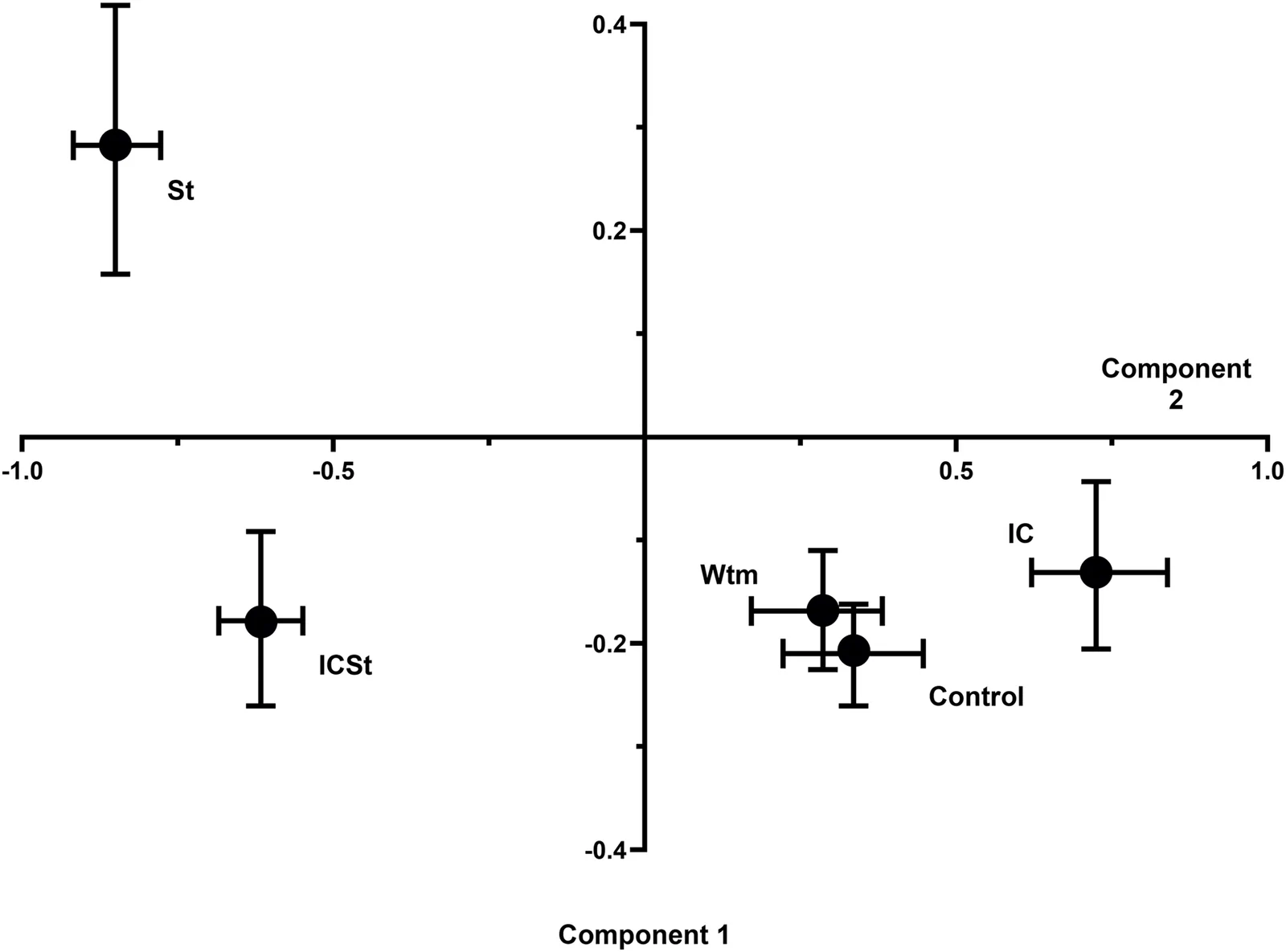
Plot of the first and second PCA components describing defensive behavioural patterns. The pattern of defensive behaviour of the starved groups was distinct from that of the other groups. Error bars represent the standard error of the mean for each component. Control (n = 33), IC – immune challenged (n = 26), St – starved (n = 26), ICSt – immune challenged and starved (n = 21), Wtm – weight matched control (n = 18). See text (Section 3.9) and Fig S5 in S1 Text for an explanation of the behavioural change.

A multivariate test of the defensive behaviours showed that they differed across groups (Pillai’s trace, F (28,464)=3.94, p < 0.001, Table 6 in S1 Text). A PCA showed that starved caterpillars had a different pattern to their defensive behaviour (Fig 17). For example, only starved caterpillars were likely to move onto the vertical rod during the trial (Fig S5E in S1 Text). Fig S5 in S1 Text shows the pattern across groups for the different defensive behaviours.

There were significant weight differences across groups on the day of testing (i.e., 5−3). (Fig 2, F(4, 101)=70.1, p < 0.001), with controls heavier than the Wtm, St and ICSt groups (Dunnett multiple comparison, p < 0.001), but there were no significant weight differences among the Wtm, St and ICSt groups (Dunnett multiple comparison test, p > 0.9). Despite the weight differences, the defensive behaviour of Wtm controls had a similar pattern to that of controls (Fig 17). These results show that the differences in defensive behaviour in IC and ICSt caterpillars are not simply a function of reduced weight and/or developmental age.

## 4 Discussion

### 4.1 Changes to the phenoloxidase pathway

Figs 1 and 11 provide a summary of the changes we observed in the phenoloxidase/eumelanin pathways during starvation. These changes suggest a shift towards a pro-inflammatory state (e.g., increased PO activity). For example, starvation led to a reduction in the abundance of the PO pathway inhibitors Serpins-5, −9 and −12 relative to Controls (Fig 11a). Serpins-5 and −12 declined in abundance in ICSt (immune challenged and starved) compared with IC (immune challenged) (Fig 11b). A decrease in Serpin-12 is likely to increase activity in the HP14 branch of the pathway ([27,69]), especially as HP14 can self-activate under certain conditions ([42,68]). We also observed an increase in the abundance of PO itself in ICSt relative to IC, and this would amplify any heightened activity in this pathway. Similarly, some key compounds in the eumelanin pathway were elevated in ICSt relative to IC caterpillars (Fig 1), despite declining resources. Dopaquinone, Dopachrome, Dopachrome conversion enzyme (DCE) and 5, 6 dihydroxyindole, (DHI) of the eumelanin pathway were all increased in abundance in ICSt relative to IC (Fig 11). Even without an immune challenge, starved caterpillars showed an increase in the abundance of Dopachrome (Fig 1, S1 Table) relative to controls. Additionally, starvation shifted activity towards the melanin arm of the eumelanin pathway (Fig 1). This arm of the pathway generates abundant ROS, making it effective for immune defense, but damaging to the host ([6]). It is this pathway that is used by the immune system ([28]). The decline in GSH during food restriction likely plays a role in producing this shift. Reduced GSH abundance leads to reduced activation of the competing pheomelanin pathway ([70]). These starvation-induced changes (Figs 1, 11) are the probable cause of the increase in spontaneous melanization in starved caterpillars (Fig 3a).

Decreasing the abundance of serpin inhibitors is a powerful method for altering the activity of the PO pathway. For example, decreasing serpins-5 and −9 in *Helicoverpa armegera* (Lepidopteran) increased PO activity and reduced susceptibility to a baculovirus infection ([71]). On the other hand, increasing serpins-5 and −9 resulted in decreased resistance ([71]). In *M. sexta*, increasing serpin-12 abundance (i.e., increasing inhibition of HP14, Fig 11) reduced PO activation ([69]). For a starving caterpillar, reducing the abundance of an inhibitor is probably less energetically expensive than increasing an activator, making it an economical method for boosting immune performance. However, reducing serpins, and other PO inhibitors such as GSH, increases the likelihood of immune-generated damage. *M. sexta* appears to increase investment in non-GSH antioxidants during starvation (Table 2), possibly to reduce the cost of the pro-inflammatory state induced by starvation. However, PO activity relies on reactive molecules for its effect ([28,29,72]). How increases in antioxidants impact PO activity is difficult to predict because of the chemical complexity of immune-oxidative chemistry in animals ([73,74]). Other insects also increase antioxidant enzymes (expression and/or production) during starvation (e.g., catalase and superoxide dismutase (SOD), *Ectomyelois ceratoniae* (Lepidoptera), [75]), suggesting that oxidative homeostasis is maintained during at least the early phases of starvation.

Additionally, the decrease in abundance of HP5, HP6, and PAP1 (Fig 11), will also reduce the connections between the PO pathway and toll pathway ([27]). The biological significance of reducing the connections between these two pathways is uncertain, but it will likely dampen the co-activation of both pathways, possibly leading to a reduction in the cost of the immune response in a starved animal.

### 4.2 Evidence for the functional significance of increased PO activity in starved caterpillars

Although starvation led to increased PO activity, other immune components were decreased. Starved caterpillars showed evidence of reduced cell-mediated immunity (i.e., reduced nodulation, Fig 13). Cellular immunity is also compromised in other insects on suboptimal diets (e.g., aphid, *Adelphorcoris suturalis*) ([76]). However, the level of starvation used in this study does not reduce hemocyte number in *M. sexta* ([19]). Therefore, the reduction in nodulation (Fig 13) probably reflects less hemocyte activity. Starvation reduced the abundance of hemocyte-specific integrin cell adhesion protein, a protein that is important for efficient cell-mediated immunity ([77]), (Fig 9), and this decline might explain some of the reduction in cell-mediated immunity. Additionally, the low glucose levels in the plasma of starved *M. sexta* (Fig 6K; [19]) might also contribute to reduced cell-mediated immunity. In *Drosophila*, hemocytes need additional glucose to activate ([1,78]).

Despite the importance of cell-mediated immunity for the control of bacterial pathogens ([22]), starved caterpillars had fewer bacteria in their hemolymph than did controls at 1h and 3h time points after the infection (Fig 12). Starved caterpillars also showed no increase in mortality for the first 48 h after infection, relative to age- and weight-matched controls. Increasing GSH in the plasma, either by injection or diet, decreased spontaneous melanization (Fig 14, 16), reducing inflammation. However, increasing GSH in starved caterpillars also led to increased mortality during a bacterial infection (*B. cereus*). These results suggest that starvation-induced inflammation (i.e., chronically elevated phenoloxidase activity) provided a benefit to starved caterpillars by enhancing early bacterial removal.

The increase in phenoloxidase/eumelanin pathway activity may help compensate for a decline in cell-mediated immunity, at least during the early phases of infection. Phenoloxidase is part of the insect’s first response to infection ([6]). Rapid clearance of bacteria is important for survival ([4]), helping to explain PO’s key role in insect immunity. The need for speed may be part of the selection pressure for starved animals to maintain mechanisms that are important for bacterial clearance early in infection. Our results suggest that starved *M. sexta* were able to rapidly remove bacteria during the early stages of infection (Fig 12a). Five min after infection we also found that the melanization rate was higher in starved caterpillars than in controls or weight-matched controls, supporting the hypothesis that increased melanization assists starved animals in maintaining bacterial removal despite the decline in nodulation. However, it should be noted that bacteria in starved animals may have a slower doubling time because of the reduced amounts of nutrients in the hemolymph, leading to an overestimation of the ability of starved caterpillars to reduce bacterial levels relative to controls. Nevertheless, the data demonstrate that starved caterpillars were able to maintain low CFU numbers during the initial phase of infection, despite declines in cell-mediated immunity.

The decline in GSH abundance during starvation appeared to contribute to the upregulation of the phenoloxidase pathway, and may be part of a suite of adaptive changes leading to increased PO activity. Altering GSH abundance in plasma is known to alter PO activity in insects. For example, increasing GSH levels reduced PO activity in *Helicoverpa zea*, ([79]) and *Pseudoplusia includens* ([35]). However, the decline in GSH abundance in starved caterpillars (Fig 6A) could also be a response to reduced resources and not the result of an adaptive process. Cysteine, one of the three amino acids in GSH, is considered limiting in insects, and GSH can consume 20% of an insects’ total cysteine ([80]). Reducing GSH could allow starving *M. sexta* to free up additional cysteine for other functions. Supplementing the diet with cysteine increased GSH abundance in the hemolymph (Fig 15), suggesting that cysteine is limiting in *M. sexta*. However, there was no significant decline in the abundance of any of the 3 amino acids in GSH (i.e., glutamate, cysteine and glycine, [80]) in the plasma of starved caterpillars (Table 1 in S1 Text). In addition, we observed a concurrent increase in the abundance of taurine and hypotaurine (Fig 6G,H), both of which contain cysteine, in ICSt caterpillars. There was also an upregulation of enzymatic protein antioxidants in both St and ICSt caterpillars (Table 2). This upregulation was probably in response to increased oxidative stress, as suggested by the raised abundance of cystine (Fig 6E + F) in St and ICSt caterpillars. It seems unlikely that making antioxidant proteins would be more resource sparing than making a tripeptide. Therefore, it seems plausible that the decline in GSH was part of the suite of biochemical network changes *M. sexta* caterpillars make when resources are short (e.g., [50,81,82]). In other words, GSH reduction may be an adaptive response to starvation, used as a mechanism to increase phenoloxidase/eumelanin pathway activity. This would explain why supplementation of starved caterpillars with GSH can be deleterious, decreasing survival after infection. Like the reduction in specific serpin inhibitors, reductions in GSH abundance could be part of an adaptive shift in immune function.

### 4.3 Final remarks

An earlier study on *M. sexta* found that 2 days of starvation led to an increase in gene expression for *attacin* and *hemolin* in the fat body ([19]). However, there was no increase in the abundance of these AMPs in the plasma of starved (St) caterpillars relative to controls (Fig 9). However, starved caterpillars given an immune challenge (ICSt) had a higher abundance of the AMPs cecropin and galectin in their plasma than did IC (Fig 10). These results suggest that transcription for AMPs may occur prior to infection in starved caterpillars. Protein production and/or release, however, does not appear to occur until a pathogen is present. We speculate that this could allow for a rapid increase in AMP abundance upon infection in starved caterpillars.

Our study took place during the active feeding period of the 5th instar, at least two days prior to dorsal vessel exposure (i.e., pre-pupal phase). During this feeding period, *M. sexta* shows a steady decline in juvenile hormone (JH) ([83]). Starvation reduces the decline in JH, such that caterpillars starved at 5th instar day 0 have about six times the amount of JH found in control caterpillars by 5th instar day 3 ([84]). JH is known to impact immune function ([83]), and the immune system of *M. sexta* is known to change over the course of the 5th instar ([36,85–87]). Therefore, starvation may induce a more ‘youthful’ immune system state. However, examination of the changes in immune function between days 5th instar 1 and 5th instar 2/3 from the literature, suggest that there is probably no significant difference in PO activity or bacterial clearance between those ages in *M. sexta* caterpillars ([86,87]). There is also no evidence for a change in the total hemocyte count during that period of the 5th instar ([85]). There was little difference in survival between 5th instar day 1 and 5th instar day 3 caterpillars injected with *Photorhabdus luminescens* ([86]) or *Stenotrophomonas maltophilia* ([87]). Therefore our results are unlikely to be caused by starved caterpillars simply being physiologically ‘younger’ than controls.

The neurohormone octopamine typically increases in plasma during stressful situations, including starvation ([66,67]). Octopamine activates the release of lipid and carbohydrates from energy stores, fueling the response to acute stressors ([66]). However, in our study, 2 days of starvation reduced octopamine plasma levels relative to controls (Fig 6I). This result is probably due to the delayed time point used in our study. For example, octopamine peaked in starved locusts (*Schistocerca gregaria*) after 8 h of starvation and then began to decline towards baseline ([88]). Similarly, octopamine typically increases in plasma during an immune challenge ([67]), but we found no evidence that octopamine was elevated in IC caterpillars relative to Controls (Fig S1 in S1 Text) at the 24 h time point. However, in earlier studies octopamine content was measured less than an hour after an immune challenge (*Periplaneta americana,* [89]; crickets, *Gryllus integer,* [90]). In cockroaches, octopamine had returned to baseline 1 h after an immune challenge ([89]). We speculate that during an extended period of starvation or infection, neurohormonal octopamine release may be inhibited to preserve dwindling energy reserves. However, the effect of starvation on neurohormonal octopamine levels demonstrates the organism-wide nature of the response ([67]). Many functions, not just immunity, are altered during starvation (e.g., muscle, [11]). In this study, the dynamics of defensive behaviour was shifted relative to controls (Fig 17). Octopamine may play a role in this shift, as it regulates many behaviours ([66,67]).

Additional research, with more time points, is also needed to determine the duration of starvation-induced inflammation. This study presents a snapshot of a dynamic process. Nevertheless, two days qualifies as chronic in this animal. Two days represents about 5% of *M. sexta*’s lifespan (approximately 40 days under our colony conditions). 5% of a 72 year human lifespan would be equivalent to 3.6 years, a time span considered chronic in humans (e.g., [91]).

Long term increases in PO activity without active infection have been reported before in insects. For example, crowding can increase phenoloxidase activity in some species (i.e., density dependent prophylaxis, [92]). This increase can improve disease resistance, but also reduce reproduction ([93]). The enhanced PO activity is thought to prepare the insect for the greater pathogen prevalence found in crowded conditions, resulting in better survival ([92]). Unfortunately, the details of how PO activity is increased by crowding remains understudied. Comparing PO activation by both crowding and starvation would help uncover some of the mechanisms regulating plasticity in the PO pathway. However, the increase in PO during starvation differs from other examples of PO upregulation in insects, because there is no increase in pathogen presence or pathogen risk. In the case of starvation, the increase in PO activity appears to be part of a reconfiguration of the immune system as opposed to a response to pathogens *per se*.

The effects of nutrition on immune function are complex in both insects and mammals (e.g., insects – [37]; mammals – [17,18]). For example, in insects, starvation can have different effects on immune function depending on whether it occurs before or after infection ([94]). Starvation after infection can enhance resistance to some pathogens in *D. melanogaster* (e.g., [39]). In *M. sexta*, illness-induced anorexia helps to resolve a physiological trade-off between digestion and immunity ([95]). Starvation prior to infection typically has negative effects on disease resistance (e.g., [96]), although the duration of the food restriction is important in determining the effect ([97]). The variation in methodology across studies (e.g., starvation duration, and starvation vs food restriction) makes comparisons difficult, but starvation/food restriction has been found to: increase (e.g., *Lymantria dispar,* [98]), decrease (e.g., *Lestes viridis,* [99], Ecol Ento 33:796, *Gryllus texensis,* [100]), or have no effect (e.g., *Pieris napi,* [101]) on PO activity. This variability suggests that whether chronic inflammation during starvation is beneficial is likely to be species-specific and context-dependent.

Nevertheless, starvation-induced inflammation is observed in both insects and mammals. Humans and rodents increase pro-inflammatory factors during starvation (e.g., an increase in C-reactive proteins and pro-inflammatory cytokines, [17,18]). During a 6 day fast in humans, biomarkers for oxidative stress and antioxidant capacity both increased ([102]), similar to what we observed in caterpillars. Starved humans also showed decreased GSH levels and increased ferritin ([103]), similar to our observations in caterpillars. Starvation also enhances some immune responses in humans (e.g., increased macrophage phagocytosis, [18]), as well as depressing others (e.g., fewer circulating B cells, [17]) depending on the duration of the starvation. Despite any positive changes on immunity, starvation leads to a reduction in disease resistance ([17]). However, the effect of reducing chronic inflammation on disease resistance in starved mammals remains relatively untested. To determine whether starvation-induced inflammation is beneficial in any animal requires comparing starved animals with chronic inflammation to starved animals in which chronic inflammation has been suppressed. Because active immune systems have costs (e.g., [1]), chronic inflammation will always appear to be less advantageous compared to healthy controls. The common practice of comparing starved animals to healthy controls is probably the main reason it has not been viewed as potentially beneficial.

As Gould and Lewontin [104] noted, not everything is adaptive; and, as ecoimmunologists know, not everything that is damaging is a pathology ([105]). We suggest that common responses to frequently faced challenges, such as food restriction, should be examined closely before being assumed to be a pathology. The interconnected nature of physiological systems means that animals are capable of reconfiguring within and across multiple organ systems to provide the best response given the circumstances ([5]). Unraveling these connections (e.g., [12,28,106]) is required to determine whether ‘damaging’ responses are maladaptive, or represent the best response given the animal’s conditions.

## Supporting information

S1 Text Supplementary methods, results, Figs S1, S2, S3, S4, S5 and S6; and Tables S3, and S6. (DOCX)

S1 Table Data set for metabolomics assay of control and starved groups. Plasma metabolite abundance data for all identified metabolites, including amino acids, across Control, and Starved (St), groups.
(XLSX)

S2 Table Data set for metabolomics assay of IC and ICSt groups. Plasma metabolite abundance data for all identified metabolites, including amino acids, across Immune challenged, and Immune challenged+ Starved (ICSt), groups.
(XLSX)

S4 Table GO annotations for identified proteins. Complete parsed proteomics dataset with Gene Ontology (GO) annotations for all proteins passing quality-control criteria (≥10% peptide sequence coverage).
(XLSX)

S5 Table List of identified immune proteins. Proteomics dataset restricted to immune-associated proteins, identified using GO analysis and UniProt annotation.
(XLSX)

S7 Table Complete metabolomics data set. Complete plasma metabolomics dataset (full, non-targeted metabolomics data underlying Figs 4–6 and Table 1).
(XLSX)
