# Supplementary material for: An upside to chronic inflammation? Starvation reconfigures immune pathways in Manduca sexta, producing chronic inflammation that may help defend against bacterial infection
Source: PLoS One. 2026 Sep 21;21(9):e0358460. doi: 10.1371/journal.pone.0358460 (PMC13592642; doi:10.1371/journal.pone.0358460)
Supplement: Supplementary file 1 [file pone.0358460.s001.docx]

**Supplementary Material**

**Supplementary Methods**

**Non-targeted Metabolomics**

Below are the details of the metabolomics methods and analysis, as provided by the Metabolomics Innovation Center at the University of Alberta.

**Sample Preparation**

Sample Randomization

Samples were randomized before any procedures to eliminate potential technical variations from sample preparation and instrument drift. The randomized samples were used for the following preparations and analyses.

Aliquoting and Generation of the Pooled Sample

Each individual sample was vortexed then centrifuged at 15,000 g for 1 min. Supernatant was taken and split into four aliquots for different labeling methods, backup and preparation of the pooled sample. For the pooled sample, 15 μL was taken from each individual sample, and then combined and mixed thoroughly, which was used as the reference.

Protein Precipitation

Each individual sample was spun down. Then, 45 μL of LC-MS grade methanol was added to perform protein precipitation. The methanol extract was completely dried after incubation at -20 °C for 0.5 hour, then temporarily stored in -80 °C freezer until labeling.

Chemical Isotope Labeling

For the one aliquot of sample for amine-/phenol-labeling, 12.5 μL of LC-MS grade water was added prior to labeling. The labeling protocol strictly followed the SOP provided in the kit. Briefly, 6.25 μL of buffer reagent (Reagent A) and 18.75 μL of ^12^C2-labeling (for the individual samples and the pooled sample) or ^13^C2-labeling (for the pooled sample) reagent (Reagent B) was added into samples. The samples were then vortexed, followed by spinning down. The mixtures were incubated at 40 °C for 45 minutes. After that, 3.75 μL of quenching reagent (Reagent C) was added to quench the excessive labeling reagent. The mixtures were incubated at 40 °C for another 10 minutes. Finally, 15 μL of pH adjusting reagent (Reagent D) was added.

For the one aliquot of sample for carboxyl-labeling, 12.5 μL of LC-MS grade ACN/water (3:1 v/v) was added prior to labeling. The labeling protocol strictly followed the SOP provided in the kit. Briefly, 5 μL of catalyzing reagent (Reagent A) and 12.5 μL of ^12^C2-labeling (for the individual samples and the pooled sample) or ^13^C2-labeling (for the pooled sample) reagent (Reagent B) was added into samples. The samples were then vortexed, followed by spinning down. The mixtures were incubated at 80 °C for 60 minutes. After that, 20 μL of quenching reagent (Reagent C) was added to quench the excessive labeling reagent. The mixtures were incubated at 80 °C for another 30 minutes and the chemical isotope labeling procedure was complete.

Mixing

The ^12^C2-labeled individual sample was mixed with ^13^C2-labeled reference sample in equal volume. The mixture was ready to be analyzed by LC-MS. Prior to LC-MS analysis of the entire sample set, a quality control (QC) sample was prepared by equal volume mix of a ^12^C-labeled and a ^13^C-labeled pooled sample.

**LC-MS Analysis Condition**

| The LC-MS analysis strictly followed the SOP (i.e., Rapid LC-MS Analysis for HP-CIL Metabolomics Platform). QC samples were injected every 16 sample runs to monitor instrument performance.  **Instrument:** | |  |
| --- | --- | --- |
| **Column:** | Thermo Scientific Vanquish LC linked to Bruker Impact II QTOF Mass Spectrometer  Agilent eclipse plus reversed phase C18 column (150 x 2.1 mm, 1.8 μm particle size) | |
| **MPA:** | 0.1% (v/v) formic acid in water | |
| **MPB:** | 0.1% (v/v) formic acid in acetonitrile | |
| **Gradient:** | t = 0 min, 25% B; t = 10 min, 99% B; t = 15 min, 99% B; t = 15.1 min, 25% B; t=18 min, 25% B | |
| **Flow Rate:** | 400 μL/min | |
| **Column Oven Temperature:** | 40 °C | |
| **Mass Range:** | m/z 220-1000 | |
| **Acquisition Rate:** | 1 Hz | |

**Data Processing**

A total of 70 LC-MS data from 2-channel analysis (35 LC-MS data, including 3 QC, in each channel) were first exported to .csv file with Bruker DataAnalysis 4.4 software. The exported data were uploaded to IsoMS Pro 1.4.0. After Data Quality Check, Data Processing was performed. Parameters used for data processing are below.

| **Minimum m/z:** | 220 |
| --- | --- |
| **Maximum m/z:** | 1000 |
| **Saturation Intensity:** | 20000000 |
| **Retention Time Tolerance** | 9 seconds |
| **Mass Tolerance:** | 10 ppm |

**Data Processing**

Five groups were assigned to 35 LC-MS data in each channel: 8 data files labeled as 'Control' group, 8 data files labeled as 'Immune Challenge' group, 8 data files labeled as 'Starved' group, 8 data files labeled as 'IC+St' group, and 3 data files labeled as 'QC' group. Peak pairs without data present in at least 80.0% of samples in any group were filtered out (see section 3.2). Data were normalized by Ratio of Total Useful Signal.

**Metabolite Identification**

| The parameters used for metabolite identification are below.  **Retention Time Tolerance for CIL Library ID** | 10 seconds |
| --- | --- |
| **Retention Time Tolerance for LI Library ID** | 75 seconds |
| **Mass Tolerance for CIL Library ID** | 10 ppm |
| **Mass Tolerance for LI Library ID** | 10 ppm |
| **Mass Tolerance for Mass-Based Database ID** | 10 ppm |

**Further Data Processing and Cleansing**

A total of 35 LC-MS data were processed for each channel. 2-channel LC-MS data of one sample were combined after data processing. Less commonly detected peak pairs were filtered out to ensure data quality. Peak pairs without data present in at least 80.0% of samples in any group were filtered out. Applying this 80%-rule ensures that only the commonly detectable peak pairs are retained for further analysis, without the use of excessive missing-value imputation. This 80% rule states that, if all the analyzed samples belong to one group, the retained peak pairs are those detectable in >= 80% of the samples. If the analyzed samples belong to two or more groups, the retained peak pairs are those detectable in >= 80% of the samples in any one of the groups.

After filtering, all data were normalized by the ratio of total useful signals. The missing values of peak pairs in some samples due to low signal intensity (i.e., below the detection limit) was replaced with a rationally determined ratio by a unique zero-imputation program. This table can be used for statistical analysis without the need of applying any missing value imputation method given in a statistical tool.

**Metabolite Identification**

Three-tier ID approach was used to perform metabolite identification.

In tier 1, peak pairs were searched against a labeled metabolite library (CIL Library) based on accurate mass and retention time. The CIL Library contains more than 1,500 experimental entries. 513 peak pairs were positively identified in tier 1.

In tier 2, linked identity library (LI Library) was used for identification of the remaining peak pairs. LI Library includes over 9,000 pathway-related metabolites, providing high-confidence putative identification results based on accurate mass and predicted retention time matches. 822 peak pairs were putatively identified in tier 2.

In tier 3, the remaining peak pairs were searched, based on accurate mass match, against the MyCompoundID (MCID) library composed of 8,021 known human endogenous metabolites (zero-reaction library), their predicted metabolic products from one metabolic reaction (375,809 compounds) (one-reaction library) and two metabolic reactions (10,583,901 compounds) (two-reaction library).

1335 peak pairs were identified as high-confidence results (tier 1 and tier 2), which were used for other analysis (e.g. pathway analysis).

**Data processing – Data Normalization**

Each metabolite is detected as a peak pair (i.e., the ^12^C-peak from individual samples and the ^13^C-peak from a pooled sample reference). For normalization, we first calculate the normalization factor by taking the ratio of the sum of the intensities of ^12^C-labeled peaks to the sum of the intensities of ^13^C-labeled peaks across all peak pairs in each sample. We then divide the peak pair ratio in each individual sample by this normalization factor. This sample-wise normalization is specifically designed for our technique and data format.

**Univariate Analysis of Metabolomics data**

Volcano plots were constructed by plotting the fold change (FC) of each metabolite against p-value. The fold change was calculated as Mean(Treatment Group) / Mean(Control). The correction for the false discovery rate was not done for the volcano plots.

**Pathway Analysis**

Comparison between the ‘Treatment’ group and 'Control' group was done using *Bombyx mori* (domestic silkworm) (KEGG) library)

Metabolites that were identified in tier 1 and tier 2 as high-confidence results were used for pathway analysis. The pathway analysis was performed using Global Test as the enrichment analysis and Relative-between ess Centrality as topology analysis in MetaboAnalyst (www.metaboanalyst.ca).

For more information, please refer to the following paper:

<https://doi.org/10.1016/j.chroma.2015.12.007>

Pathway analysis followed the methods used by MetaboAnalyst ( <https://academic.oup.com/bioinformatics/article/26/18/2342/208464?login=true>). Note that the pathway analysis assesses the magnitude of the difference between metabolites in a given pathway between two groups. It does not assess the direction of the change.

The pathway impact is calculated as the sum of the importance measures of the matched metabolites normalized by the sum of the importance measures of all metabolites in each pathway. (https://academic.oup.com/bioinformatics/article/26/18/2342/208464?login=true). Xia and Wishart, 2010. MetPA: a web-based metabolomics tool for pathway analysis and visualization. Bioinformatics. 26 (8): 2342-2344. <https://doi.org/10.1093/bioinformatics/btq418>

**Metabolomic box plots**

The box plots (Fig. 8) summarize the distribution of data for the two compared groups (e.g., Starvation vs. Controls). The x-axis of the plot is group information (i.e., unitless). The y-axis represents the log_2_ ratio of the peak intensity ratio (i.e., ^12^C-samples/^13^C-reference) for the corresponding metabolite. The units cancel out when calculating these ratios. q-value is the false discovery rate adjusted p-value, which is used in the multiple hypothesis testing.

**2. Qlucore Data Analysis**

Calculation of False Discovery Rate (FDR and q-value)

For details see: (Benjamini and Hochberg, 1995): Controlling the False Discovery Rate: A Powerful and Practical Approach to Multiple Testing. Journal of the Royal Statistical

Society Series B 57 (1995), 289-300.

Heat map colours reflect the value for each sample for each variable (i.e. metabolite or protein). The colour scale is adjusted using a Z-score to minimize the impact of potential outliers. The colour scale does not reflect the entire range of data values. Fig S6 demonstrates the colour scale used in the heat maps. Exact values are available in Tables S1 (metabolites) and Tables S3/S4 (proteins).

**3. GO Analysis Methods**

The GO Analysis was conducted using PANTHER (<https://pantherdb.org>). The *M. sexta* proteins were mapped to *Drosophila melanogaster* genes using the accession numbers from Uniprot (uniport.org) and converting these to gene ID numbers. This was done by searching up the proteins using the NCBI gene database using NCBI Orthology and Orthologs from OrthoDB and NCBI BLASTp. The pie charts and GO Trees were made using the differentially abundant proteins between the different groups. The differentially expressed proteins were set against controls on a g:Profiler using a g:GOSt (used for functional profiling). The significance threshold was set using the Benjamini-Hochberg FDR procedure (q<0.05).

**4. Targeted Mass Spectrometry Analysis**

Methods were supplied by the Biology Mass Spectrometry Centre at Dalhousie University.

Metabolites were extracted from an aliquot of 50µL of plasma using Captiva EMR-Lipid wellplates (Agilent Technologies) on a vacuum manifold. Prior to extraction, each sample was spiked with a mixture of isotopically labeled amino acids (Cambridge Isotopes, CAT#MSK-A2-1.2). EMR wells were preconditioned using 600µL of a mixture of 2:1:1 water/methanol/ethanol. The plasma aliquot was added to the wells and 225µL of 1:1 methanol/ethanol was added and pipette mixed with incubation for 10-minutes at room temperature. To each well, 225µL of water was added and the supernatant filtered through the well and the flowthrough was collected. Wells were rinsed twice with 2:1:1 water/methanol/ethanol and combined with the previous flowthrough. Samples were dried in a SpeedVac and reconstituted in 60% acetonitrile in water prior to mass spectrometry analysis. Targeted mass spectrometry analysis was carried out on a QTRAP5500 instrument interfaced to an Agilent 1290 Infinity II liquid chromatography (LC) system controlled using Analyst software (version 1.6.3). For analysis, samples were directly separated by an analytical column (2.1mm x 150mm, ACQUITY Premier BEH Amide 1.7µm, Waters) at a flow rate of 0.25mL/min. For analyte elution the gradient held at an initial starting condition of 100% mobile phase B (95% acetonitrile, 5% water, 5mM ammonium acetate, 5mM ammonium hydroxide) for 2-minutes before ramping to 10% B over 2.5 minutes and holding for 1-minute, ramping to 100% B over 0.5-minutes with a final equilibration at 100% B for 4-minutes (total run time of 10-minutes, mobile phase A = 95% water, 5% acetonitrile, 5mM ammonium acetate, 5mM ammonium hydroxide). Data acquisition on the QTRAP5500 utilized a targeted selected reaction monitoring (SRM) acquisition scheme at 'unit' resolution in both positive and negative polarity modes. Positive mode used a curtain gas setting of 20, CAD gas of 5, ion spray voltage of 5500V, temperature of 350, gas 1 of 55, gas 2 of 60, declustering potential of 100, entrance potential of 10, and cell exit potential of 20. Negative mode used the same settings as positive with the exceptions of ion spray voltage of -4500V, declustering potential of -100, entrance potential of -10, cell exit potential of -20. Method transitions given below.

| Compound | Q1 mass | Q3 mass | Dwell time | CE | Polarity |
| --- | --- | --- | --- | --- | --- |
| cysteine | 122.03 | 105.01 | 20 | 10 | positive |
| cysteine | 122.03 | 86.98 | 20 | 15 | positive |
|  |  |  |  |  |  |
| glutathione | 308.09 | 179.04 | 20 | 15 | positive |
| glutathione | 308.09 | 162.01 | 20 | 20 | positive |
| glucose | 179.06 | 89.01 | 20 | -10 | negative |
| glucose | 179.06 | 70.99 | 20 | -15 | negative |

| trehalose | 341.11 | 89.01 | 20 | -25 | negative |
| --- | --- | --- | --- | --- | --- |
| trehalose | 341.11 | 59.01 | 20 | -45 | negative |

(list: metabplasmaq<0.05listalltreatsept30 in metabplasmafinalsept30 folder in uniqlucore – check wth NB: use metabdataandfigsapr2026 in Mandimminflam… folder.).

**Supplementary Results**

**1. Metabolomics**

Complete data set from Metabolomics is in Table S7.

Fig S1. Heat map of the identified metabolites that are significantly different (i.e. at least a 2 fold change, q<0.05) in abundance in the plasma of immune challenged (IC) caterpillars relative to Controls. Red squares demonstrate higher abundance, while blue squares represent lower abundance. The metabolite is identified along the right of the heatmap. The darkness of the colour reflects the amplitude of the increase or decrease. Scale color bar is shown in Fig S6. N=8 for each group.

**Significant Pathway Alterations**

**Table S3**

To maintain statistical power, only *a priori* comparisons were made. Namely:

Control vs starved (St); control vs immune challenge (IC), and IC vs ICSt. Only pathways with impact >0.5 and FDR adjusted p-value ≤0.05 are listed.

*Control vs Starved*

| **Pathway Name** | **Total Cmpd** | **Hits** | **Raw p-value** | **Holm adjust p-value** | **FDR adjusted p-value** | **Impact** |
| --- | --- | --- | --- | --- | --- | --- |
| Arginine and proline metabolism | 29 | 18 | 1.77E-06 | 8.32E-05 | 2.21E-05 | 0.92022 |
| beta-Alanine metabolism | 16 | 8 | 5.99E-06 | 0.00026345 | 4.28E-05 | 0.69381 |
| Cysteine and methionine metabolism | 34 | 18 | 2.33E-05 | 0.00097885 | 0.00012933 | 0.77362 |
| Glycine, serine and threonine metabolism | 30 | 15 | 2.85E-05 | 0.0011412 | 0.00012933 | 0.70905 |
| Tryptophan metabolism | 29 | 14 | 5.04E-05 | 0.0019637 | 0.00019871 | 0.58973 |
| One carbon pool by folate | 23 | 14 | 6.09E-05 | 0.0022541 | 0.00021757 | 0.70985 |
| Alanine, aspartate and glutamate metabolism | 21 | 13 | 9.97E-05 | 0.0035894 | 0.00032704 | 0.51272 |
| Glutathione metabolism | 26 | 10 | 0.00011119 | 0.0037806 | 0.00032704 | 0.5204 |

*Control vs Immune Challenge*

Top change listed below, but none significant

| **Pathway Name** | **Total Cmpd** | **Hits** | **Raw p-value** | **Holm adjust p-value** | **FDR adjusted p-value** | **Impact** |
| --- | --- | --- | --- | --- | --- | --- |
| Glycine, serine and threonine metabolism | 30 | 15 | 0.003281 | 0.16077 | 0.082024 | 0.70905 |

*Immune Challenge vs Immune Challenge and Starvation*

| **Pathway Name** | **Total Cmpd** | **Hits** | **Raw p-value** | **Holm adjust p-value** | **FDR adjusted p-value** | **Impact** |
| --- | --- | --- | --- | --- | --- | --- |
| Cysteine and methionine metabolism | 34 | 18 | 2.03E-08 | 1.02E-06 | 5.35E-07 | 0.77362 |
| Arginine and proline metabolism | 29 | 18 | 2.14E-08 | 1.05E-06 | 5.35E-07 | 0.92022 |
| Glycine, serine and threonine metabolism | 30 | 15 | 5.10E-06 | 0.00023957 | 4.82E-05 | 0.70905 |
| Taurine and hypotaurine metabolism | 7 | 4 | 5.78E-06 | 0.00026007 | 4.82E-05 | 1 |
| Arginine biosynthesis | 13 | 9 | 3.10E-05 | 0.0013039 | 0.00017248 | 0.90908 |
| One carbon pool by folate | 23 | 14 | 7.97E-05 | 0.0030274 | 0.00030642 | 0.70985 |
| Alanine, aspartate and glutamate metabolism | 21 | 13 | 0.00028426 | 0.010234 | 0.00094755 | 0.51272 |
| Tryptophan metabolism | 29 | 14 | 0.0010788 | 0.033443 | 0.0026971 | 0.58973 |
| Beta-Alanine metabolism | 16 | 8 | 0.0014817 | 0.043059 | 0.0032211 | 0.69381 |
| Glutathione metabolism | 26 | 10 | 0.0014392 | 0.043059 | 0.0032211 | 0.5204 |

**2. Proteomics**

Fig. S2. GO Tree for differentially abundant proteins (102 proteins) between Starved and Control caterpillars n=10/group. Highlighted boxes are the most enriched central GO terms. The boxes with a yellow border represent GO terms that are significantly over-represented, after Benjamini-Hochberg FDR correction, compared with the background protein list.

Fig. S3. GO Categorization of the Molecular Functions for differentially abundant proteins between IC (immune-challenged) and ICSt (immune-challenged and starved) caterpillars. n=10/group. There were 183 differentially abundant proteins in IC relative to ICSt.

Fig S4. GO characterization of the Biological Processes for differentially abundant proteins between IC (immune-challenged) and ICSt (immune-challenged and starved) caterpillars. n=10/group. There were 183 differentially abundant proteins in IC relative to ICSt.

**3. Defensive Behaviour**

**Table S6**

**Multivariate Analysis of Defensive Behavior in Response to Tactile Stimulation.**

**Statistical Summary Tables.**

| **Tests of Between-Subjects Effects** | | | | | | | | | |
| --- | --- | --- | --- | --- | --- | --- | --- | --- | --- |
| Source | Dependent Variable | Type III Sum of Squares | df | Mean Square | F | Sig. | Partial Eta Squared | Noncent. Parameter | Observed Power^h^ |
| Corrected Model | Single Defensive Strike | 3578.459^a^ | 4 | 894.615 | 11.481 | <.001 | .278 | 45.925 | 1.000 |
|  | Repeated Defensive Strikes | 1078.576^b^ | 4 | 269.644 | 9.715 | <.001 | .246 | 38.860 | 1.000 |
|  | Use of Mandibles | 3656.131^c^ | 4 | 914.033 | 11.125 | <.001 | .272 | 44.500 | 1.000 |
|  | Retraction of the A6 Leg | 1585.699^d^ | 4 | 396.425 | 8.345 | <.001 | .219 | 33.379 | .998 |
|  | Climbing the Vertical Rod | 69.482^e^ | 4 | 17.371 | 3.262 | .014 | .099 | 13.049 | .822 |
|  | Partial Strike | 30.929^f^ | 4 | 7.732 | 4.794 | .001 | .139 | 19.175 | .948 |
|  | No Response | 216.832^g^ | 4 | 54.208 | 9.395 | <.001 | .240 | 37.580 | .999 |
| Intercept | Single Defensive Strike | 21085.711 | 1 | 21085.711 | 270.611 | <.001 | .695 | 270.611 | 1.000 |
|  | Repeated Defensive Strikes | 1639.998 | 1 | 1639.998 | 59.087 | <.001 | .332 | 59.087 | 1.000 |
|  | Use of Mandibles | 17585.134 | 1 | 17585.134 | 214.036 | <.001 | .643 | 214.036 | 1.000 |
|  | Retraction of the A6 Leg | 3107.762 | 1 | 3107.762 | 65.418 | <.001 | .355 | 65.418 | 1.000 |
|  | Climbing the Vertical Rod | 57.791 | 1 | 57.791 | 10.853 | .001 | .084 | 10.853 | .905 |
|  | Partial Strike | 41.441 | 1 | 41.441 | 25.692 | <.001 | .178 | 25.692 | .999 |
|  | No Response | 1314.707 | 1 | 1314.707 | 227.858 | <.001 | .657 | 227.858 | 1.000 |
| group | Single Defensive Strike | 3578.459 | 4 | 894.615 | 11.481 | <.001 | .278 | 45.925 | 1.000 |
|  | Repeated Defensive Strikes | 1078.576 | 4 | 269.644 | 9.715 | <.001 | .246 | 38.860 | 1.000 |
|  | Use of Mandibles | 3656.131 | 4 | 914.033 | 11.125 | <.001 | .272 | 44.500 | 1.000 |
|  | Retraction of the A6 Leg | 1585.699 | 4 | 396.425 | 8.345 | <.001 | .219 | 33.379 | .998 |
|  | Climbing the Vertical Rod | 69.482 | 4 | 17.371 | 3.262 | .014 | .099 | 13.049 | .822 |
|  | Partial Strike | 30.929 | 4 | 7.732 | 4.794 | .001 | .139 | 19.175 | .948 |
|  | No Response | 216.832 | 4 | 54.208 | 9.395 | <.001 | .240 | 37.580 | .999 |
| Error | Single Defensive Strike | 9272.340 | 119 | 77.919 |  |  |  |  |  |
|  | Repeated Defensive Strikes | 3302.908 | 119 | 27.756 |  |  |  |  |  |
|  | Use of Mandibles | 9776.990 | 119 | 82.160 |  |  |  |  |  |
|  | Retraction of the A6 Leg | 5653.228 | 119 | 47.506 |  |  |  |  |  |
|  | Climbing the Vertical Rod | 633.639 | 119 | 5.325 |  |  |  |  |  |
|  | Partial Strike | 191.942 | 119 | 1.613 |  |  |  |  |  |
|  | No Response | 686.612 | 119 | 5.770 |  |  |  |  |  |
| Total | Single Defensive Strike | 33937.000 | 124 |  |  |  |  |  |  |
|  | Repeated Defensive Strikes | 6430.000 | 124 |  |  |  |  |  |  |
|  | Use of Mandibles | 32139.000 | 124 |  |  |  |  |  |  |
|  | Retraction of the A6 Leg | 11023.000 | 124 |  |  |  |  |  |  |
|  | Climbing the Vertical Rod | 767.000 | 124 |  |  |  |  |  |  |
|  | Partial Strike | 258.000 | 124 |  |  |  |  |  |  |
|  | No Response | 2279.000 | 124 |  |  |  |  |  |  |
| Corrected Total | Single Defensive Strike | 12850.798 | 123 |  |  |  |  |  |  |
|  | Repeated Defensive Strikes | 4381.484 | 123 |  |  |  |  |  |  |
|  | Use of Mandibles | 13433.121 | 123 |  |  |  |  |  |  |
|  | Retraction of the A6 Leg | 7238.927 | 123 |  |  |  |  |  |  |
|  | Climbing the Vertical Rod | 703.121 | 123 |  |  |  |  |  |  |
|  | Partial Strike | 222.871 | 123 |  |  |  |  |  |  |
|  | No Response | 903.444 | 123 |  |  |  |  |  |  |
| a. R Squared = .278 (Adjusted R Squared = .254) | | | | | | | | | |
| b. R Squared = .246 (Adjusted R Squared = .221) | | | | | | | | | |
| c. R Squared = .272 (Adjusted R Squared = .248) | | | | | | | | | |
| d. R Squared = .219 (Adjusted R Squared = .193) | | | | | | | | | |
| e. R Squared = .099 (Adjusted R Squared = .069) | | | | | | | | | |
| f. R Squared = .139 (Adjusted R Squared = .110) | | | | | | | | | |
| g. R Squared = .240 (Adjusted R Squared = .214) | | | | | | | | | |
| h. Computed using alpha = .05 | | | | | | | | | |

**A)**

**B)**

**C)**

**D)**

**E)**

**F)**

**G)**

Figure S5. The frequency of different defensive behaviours by group (Control, IC (immune-challenged), St (Starved), ICSt (Immune challenged and starved), Wtm (weight matched control). A) Full Strike. B) Repeated Strike. C) Mandible Use. D) Retraction of A6 Leg. E) Climbing the Vertical Rod. F) Partial Strike. G) No Response. The central bar line denotes the median, with the top of the bar representing the 75^th^ percentile, and the bottom of the bar representing the 25^th^ percentile. Error bars show the maximum and minimum values.

Fig S6. Scale bar for heat maps (Figs. 7, 8, 9, 10, FigS1). Heat map colors reflect the value for each sample for each variable (i.e. metabolite or protein). The color scale is adjusted using a Z-score to minimize the impact of potential outliers. The color scale does not reflect the entire range of data values. Exact values are available in Tables S1 (metabolites); Tables S3 and S4 (proteins).
